# Shorter headed dogs, visually cooperative breeds, younger and playful dogs form eye contact faster with an unfamiliar human

**DOI:** 10.1038/s41598-021-88702-w

**Published:** 2021-04-29

**Authors:** Zsófia Bognár, Dóra Szabó, Alexandra Deés, Enikő Kubinyi

**Affiliations:** Senior Family Dog Project, Department of Ethology, Eötvös Loránd University, Budapest, Hungary

**Keywords:** Zoology, Animal behaviour

## Abstract

Forming eye contact is important in dog–human communication. In this study we measured what factors affect dogs’ propensity for forming eye contact with an experimenter. We investigated the effect of [1] cephalic index (head shape’s metric, indicator of higher visual acuity at the centre of the visual field), [2] breed function (visual cooperativeness), [3] age and [4] playfulness with strangers in 125 companion dogs. Cephalic index was measured individually and analysed as a continuous variable. Results showed that [1] dogs with a higher cephalic index (shorter head) established eye contact faster. Since cephalic index is highly variable even within a breed, using artificial head shape groups or breed average cephalic index values is not recommended. [2] Breed function also affected dogs’ performance: cooperative breeds and mongrels established eye contact faster than dogs from non-cooperative breeds. [3] Younger dogs formed eye contact faster than older ones. [4] More playful dogs formed eye contact faster. Our results suggest that several factors affect dogs’ interspecific attention, and therefore their visual communication ability.

## Introduction

Dogs are well adapted to living with humans, partly due to their effective use of human communicative signals. Humans predominantly use eye contact to establish communication^1^, and dogs are sensitive to this cue (e.g. they follow human pointing^2^ and gaze^3^ more successfully if eye contact is established prior to the presentation of the cue). Dogs’ increased attention to humans enhances the effectiveness of dog–human communication and thus cooperation. Gaze direction can moreover be considered as an indicator of attentional focus^4^. Mutual gaze also plays a role in dog–human bonding. Its duration is associated with increased oxytocin levels in both dogs and their human partners^5^. As eye contact plays a fundamental role in dog–human relationships, it is important to know which factors influence it.

The amount of variation in the head shape of modern dog breeds is unique^6^. Relationships have been found between dogs’ head morphology, brain organization and sensory abilities^7–11^. For instance, there is an indirect connection between head shape and dogs’ visual acuity^10^. Head shape can be measured objectively with its common metric, the *cephalic index*^12^, a ratio of the width and the length of the head. The *cephalic index* is correlated with the distribution of the eyes’ retinal ganglion cells. These cells are responsible for the initial pre-processing of visual information from retinal photoreceptors. There is a difference between long-headed (*dolichocephalic*; low *cephalic index* value) and short-headed (*brachycephalic*; high *cephalic index* value) dogs with respect to the retinal ganglion cells’ distribution. In the case of *dolichocephalic* dogs, these cells form a horizontally aligned visual streak, while in *brachycephalic* dogs the cells have a higher density at the centre of the field of vision and lower in the periphery^10^. As a likely consequence, *brachycephalic* dogs may be better able to focus their attention to stimuli at the centre of their visual field, where their communication partner is situated, because they are less disturbed by other visual stimuli coming from the periphery. As a result, they may display a better visual communication ability. Gácsi et al.^13^ found that *brachycephalic* dogs are more successful at following humans’ visual gestures than *dolichocephalic* dogs. To detect these cues, the animals need to look at the human’s upper body, thus *brachycephalic* dogs may be also more prone to form eye contact with humans. In line with this, Bognár et al.^14^ showed that *brachycephalic* dogs watch motionless, projected faces of both dogs and humans over a longer time than *dolichocephalic* dogs. Taken together, these above-mentioned differences between *brachycephalic* and *dolichocephalic* dogs suggest that *cephalic index* may be linked with changes in the way dogs perceive stimuli and possibly process information, and hence with differences in canine behaviour and social cognition.

**Figure 1:**
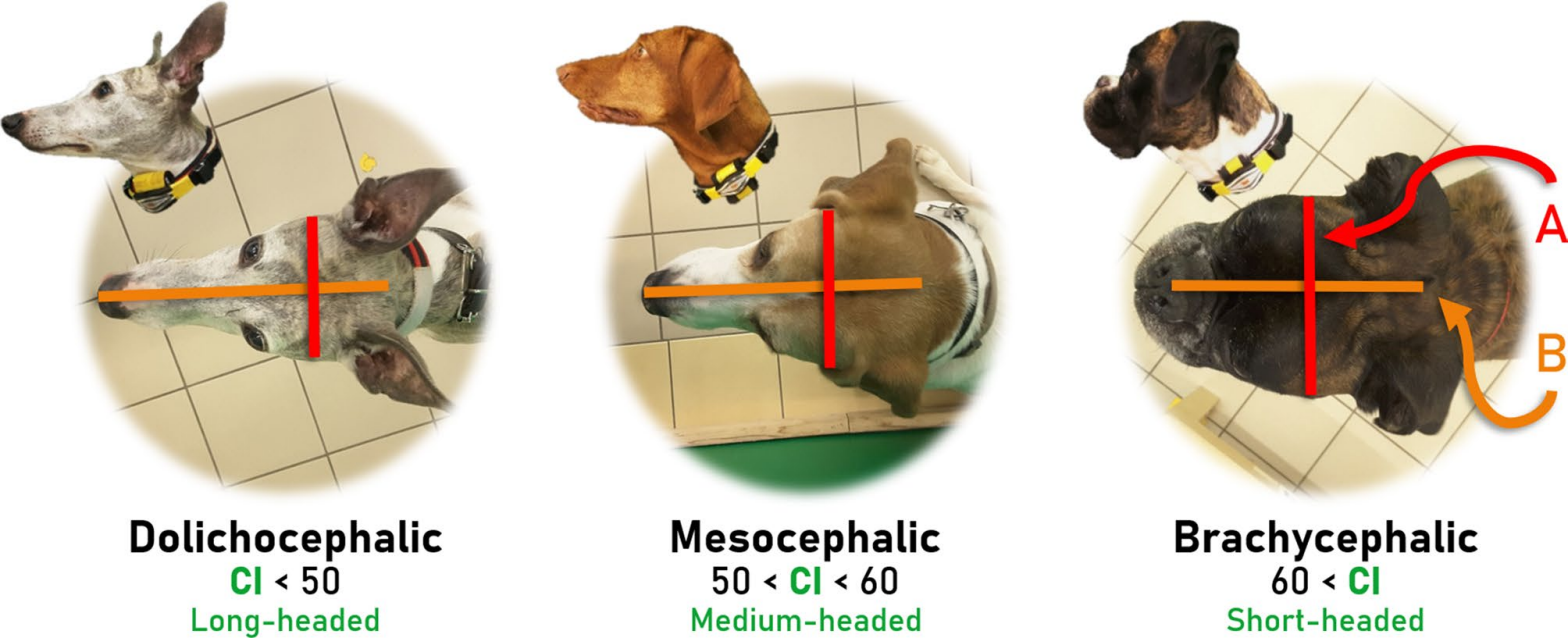
The typical classification of dogs’ head shape based on the *cephalic index* value. *Cephalic index* (CI) is the ratio of the maximum width of the head (**A**) multiplied by 100 divided by the head’s maximum length (**B**). The shorter a dog’s head is, the higher the cephalic index.

The typical classification of dogs’ head shape based on cut-off values (Fig. 1) has been criticized by Georgevsky et al.^15^ as arbitrary. The *cephalic index* is a continuous variable with no sharply separable thresholds, and the value can vary over a wide range even within a breed (see Stone et al.^16^). Therefore, we decided to study the effect of head shape using continuous *cephalic index* values instead of threshold-based grouping, and at an individual level instead of using breed averages. This way we do not have to exclude mongrels, for which a breed average cannot be calculated.

As morphological characteristics may not be independent of a breed’s function, it should also be taken into account. During breeding, dogs were selected for different types of work, which may have modified their communication skills. Consequently, not all breeds pay attention equally to human visual cues^17–21^. One possible way to group dog breeds is based on the role of vision in their cooperation with a human partner. Visually *cooperative breeds* have been selected to work in continuous visual contact and interaction (e.g. herding dogs), thus they are expected to be attentive to humans. On the contrary, visually *non-cooperative breeds* are not in visual contact with the human during their work (for example sled dogs and hounds), so they are not expected to pay as close attention to a human as *cooperative* ones. Previous research found that *cooperative breeds* are more successful at following human pointing gestures than *non-cooperative breeds* and *mixed breed dogs*^13^. On the other hand, we found no difference between breed types in their attention to motionless, projected faces of both dogs and humans^14^. Selection for different functions and head shape are not independent, but the relationship between them is not exclusive^15^. Dogs with low and high *cephalic index* value can be found among both *cooperative* and *non-cooperative breeds*. Based on this, breed function and head shape can cumulatively affect the visual communication ability of dogs.

Aging also significantly impacts visual attention^22–31^. A generalised slowing of information processing provides an explanation for an overall age-related decline in cognition^25^. Both an age-related reduction in visual processing speed^26^ and a decrease in visual contrast sensitivity have been reported in the human literature^32–34^, which can affect face perception^32,35,36^. Reduced visual processing speed could also result in a decrease in aged dogs’ social attention, which could hinder cooperation and communication between dogs and humans. Several previous studies showed an age-related decrease in visual^24,31^ and social attention^27–30^ (e.g. reported behavioural signs in older dogs included a reduction in attention towards the owner, and declines in play related activities etc.). Wallis et al.^27^ examined the effect of aging on Border collies’ (aged from 6 months to 14 years) propensity to form eye contact with an unfamiliar human. They found a quadratic relationship between propensity and age, and the performance peaked in middle aged dogs (3–6 years old animals established eye contact the fastest). Later Chapagain et al.^24^ expanded this to other dog breeds in a sample of dogs aged above 6 years, and found no effect of aging. As head shape and breed function may affect visual communication ability in dogs, to study the effect of aging, these factors should be accounted for.

Additionally, dogs’ propensity to form eye contact with an unfamiliar human can also be influenced by sociability (friendliness toward strangers^16^). Jakovcevic et al.^37^ reported that more sociable dogs gaze longer at a human face, than less sociable ones.

Since previous studies compared arbitrary head shape groups and/or the *cephalic index* was averaged by breed, currently, it is not possible to determine whether the differences in association with head shape that were found are due to breed differences or if they can be detected also independent of breed (e.g., by comparing individuals with different CI within breeds). Since *cephalic index* varies in a high range even within a breed^16^, it would be worthwhile to measure it individually, and also analyse its connections individually. The aim of this study was to consider the possible influencing factors that affect dogs’ human-directed attention and the visual communication ability in order to better understand their complex interplay, and to investigate whether head shape, breed function, age and/or sociability show any association with the individual’s propensity to form eye contact with humans.

In this study, we replicated the test of Wallis et al.^27^ and Chapagain et al.^24^, to measure pet dogs’ propensity to form eye contact with an unfamiliar experimenter, who rewarded the dog for repeatedly making eye contact with her. We investigated whether *cephalic index* is connected with this propensity, also taking into account other important influencing factors, such as breed function, age and sociability with humans. In order to gather data on the sociability of dogs, we tested their interest in a stranger. We examined the extent of greeting behaviour towards an unfamiliar human, and, as dogs' playfulness with humans and their interest in strangers are closely connected^38^, we also tested their playing behaviour towards the experimenter. We hypothesized that:

1. The *cephalic index* is positively correlated with dogs’ propensity to make eye contact with a human, so that shorter headed dogs (with a higher *cephalic index*) would form eye contact faster.
2. Visually *cooperative breeds* would form eye contact faster than *non-cooperative breeds* and *mixed breed dogs*.
3. Older dogs would form eye contact slower than younger dogs.
4. More social dogs would form eye contact faster with the unfamiliar person than less social ones.

## Methods

### Ethical statement

The Animal Welfare Committee of Eötvös Loránd University approved and accepted the experimental protocol (Ref. no.: PE/EA/2019-5/2017) and the tests were performed in accordance with the Hungarian regulations on animal experimentation and the Guidelines for the use of animals in research described by the Association for the Study Animal Behaviour (ASAB) and ARRIVE.

### Subjects

In this study 130 pet dogs were tested, from which five had to be excluded: (1) because of problems with the video (N = 1), (2) visibility of dog’s eyes (due to coat N = 1), (3) problems with eating the food from the ground because of mouth morphology (N = 2) and (4) insufficient food motivation (N = 1). Wallis et al.^27^ found that the peak of dogs’ performance in eye contact forming with humans is at middle age, and we were interested in aging, not maturation, hence we only tested dogs older than 2.5 years. Thus, 125 dogs (male = 62) were included in the analysis (*cephalic index* value: 43.5–74.7 (median = 53.2); age: 31.4–174.5 months (median = 106.5 months).

The grouping of the dogs into breed functions was based on Gácsi et al.^13^ and the dogs’ breed history. The *Cooperative breed* group (N = 42) contained breeds which have been selected to work in continuous visual contact and interaction with a human partner (e.g. sheepdogs, gundogs), in contrast to the *Non-cooperative breed* group (N = 27; e.g. hounds, sled dogs, guard dogs, earthdogs). The *Mixed breed* group (N = 56) consisted of non-purebred dogs with unknown ancestors. Owners provided information about how did they get their dog; 68% of *mixed breed* dogs in our sample (38/56) were adopted from an animal shelter or found on the street, while only 7% of them (4/56) were adopted from a previous owner, and 16% of them (9/56) were gifted to the actual owner (with no information on whether they were found on the street or rescued from a shelter). The origin of 9% of *mixed breed*s (5/56) was totally unknown. It is unlikely, that the *mixed breed* dogs in our sample were first-generation mixtures, but testing the *mixed breed* dogs' lineages goes beyond the aims of the present study.

Neither the distribution of *cephalic index* nor the distribution of age differed between the breed groups (see Table 1). *Cephalic index* value and age did not correlate (R = 0.137; *p* = 0.129). All subjects’ demographic data are presented in Supplementary Table S1.

**Table 1:** *Cephalic index* and age distribution of the sample among the different breed function groups.

	Cooperative	Non-cooperative	Mixed	Statistics
Cephalic index (mean ± SD)	53.28 ± 5.28	53.84 ± 8.27	53.57 ± 5.14	F_2,122_ = 0.075; *p* = 0.928
Age (month, mean ± SD)	107.70 ± 38.10	89.27 ± 37.90	101.30 ± 39.87	F_2,122_ = 1.850; *p* = 0.162

Dogs can be taught to form eye contact with the owner. In several dog schools, one of the first tasks is to teach the dog to make eye contact and thereby increase attention to the owner. We have data on the previous dog school experience (yes/no) from only 113 dogs. 73 dogs attended dog school, while 40 dogs did not. Dog school attendance had no connection to dogs’ performance in our test (hazard ratio = 1.049; *p* = 0.847).

### Cephalic index coding

As mentioned in the introduction, the typical classification of dogs’ head shape based on cut-off values (Fig. 1) has been criticized by Georgevsky et al.^15^ due the arbitrary nature of the grouping, thus we studied the effect of head shape with actual *cephalic index* values. We measured each dog’s *cephalic index* from photographs. The method of measuring *cephalic index* from photographs was suggested by previous studies^11,16,39^. The *cephalic index* value was measured from photographs with the GIMP image editing program 2.2.13. (http://www.gimp.org/). The index was calculated as the ratio of the maximum width of the head multiplied by 100 divided by the head’s maximum length (Fig. 1). Skull width was measured from one zygomatic arch to the other and skull length was measured from the nose to the occipital protuberance. Each picture was taken from the same angle (perpendicular to the top of the skull; see examples in Fig. 1). The distance of the camera (Samsung T710 Galaxy Tab S2) to the top of the dogs’ head was not uniform (as each dog was a different height, and the camera was not fixed), however, this did not affect the measurement, as cephalic index is a ratio. To check the reliability of measuring the *cephalic index* from photographs, a second coder, naïve to the hypotheses of the study, measured a random sample of subjects (~ 20% of dogs; ICC: 0.91, *p* < 0.001), and in addition, the heads of ~ 20% of the dogs were also measured with a calliper (ICC: 0.98, *p* < 0.001). In the case of a Puli, measuring the *cephalic index* from a photograph was not possible because of its hair, thus it was measured only with a calliper (the dog’s hair was tied up during the test, so its eyes were visible).

### Behaviour tests

All dogs participated in the “Canine Cognitive Battery” (Kubinyi et al., in prep.), which consists of 12 subtests. The prerequisite of participation was to meet the requirements of a sensory examination^40^. The same experimenter performed all subtests for an individual dog; however, the experimenter identity could differ between dogs. The first test where the dog met with the unfamiliar experimenter was the *Greeting* test, which was immediately followed by the *Human-directed play* test. The *Eye contact establishment* test was the tenth subtest. Thus, before the *Eye contact establishment* subtest, all dogs had prior experience with the experimenter, who positively interacted with the dog (stroking, playing, speaking, and feeding them). As the participating dogs were enrolled in our longitudinal research project and it was important for the experimenter to be unfamiliar to the dog at the beginning of the *Greeting* subtest, 8 experimenters took part in testing to comply with this requirement (the experimenter’s ID can be found in Supplementary Table S1). All experimenters were young women (age: 20–27 years). All three tests were carried out during one test session in the same laboratory room (6.27 m * 5.4 m).

### Eye contact establishment test

**Figure 2:**
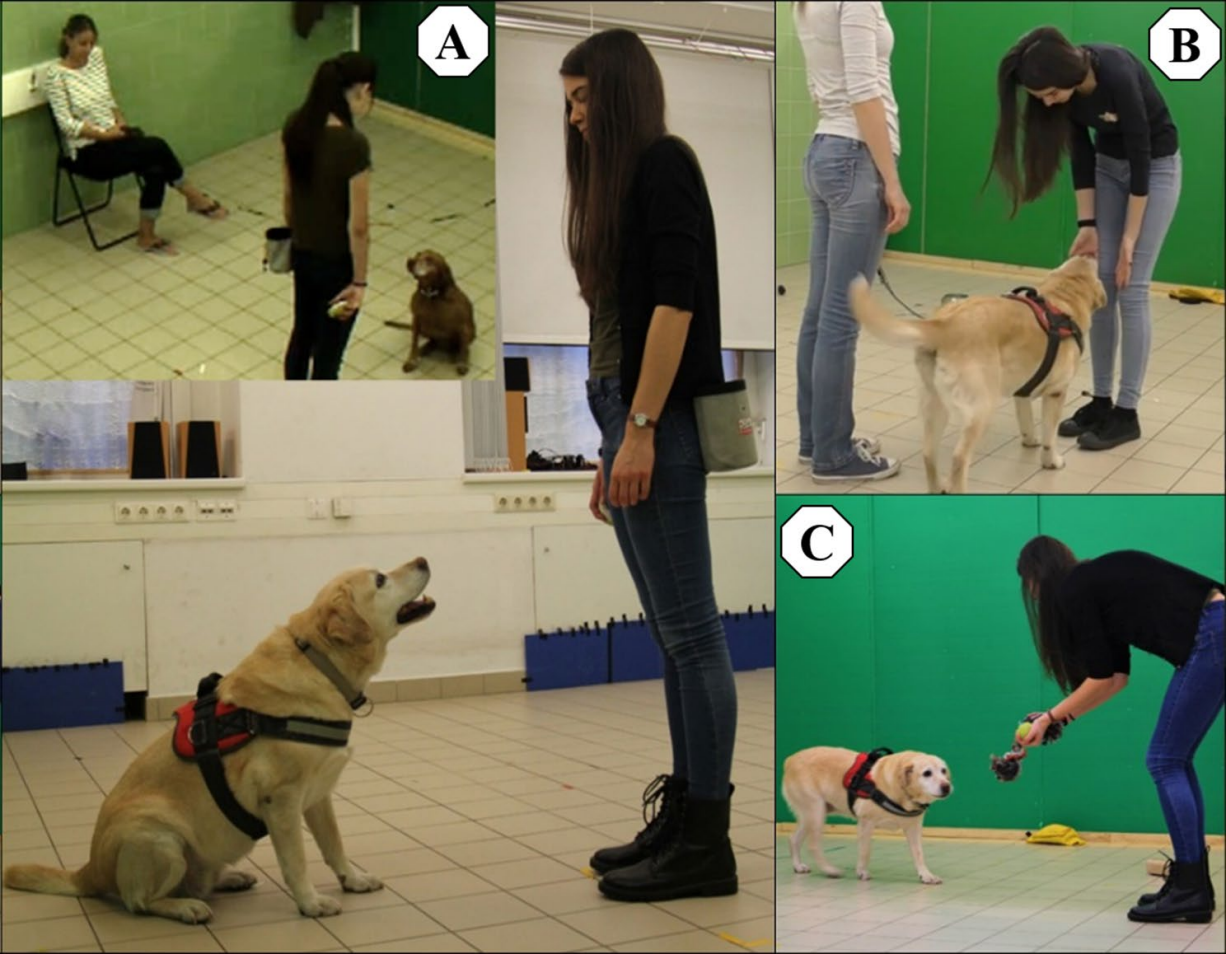
Test setup. *Eye contact establishment* test (**A**), *Greeting* test (**B**) and *Human-directed play* test (**C**).

In this test, which was based on Wallis et al.^27^ and Chapagain et al.^24^, dogs were rewarded for repeatedly forming eye contact with the experimenter. During the test, the experimenter stood in the centre of the laboratory room, while the owner sat on a chair (Fig. 2A). The experimenter held a clicker-like device (which made a “*boing*” sound, different from the usual clicker sound) in one of her hands, while the other hand was free. During the test, both hands were in a relaxed position by her side. She also had a food pouch at her back on her belt containing pieces of sausage as a food reward.

At the beginning of the test, the experimenter called the dog to her, and threw a piece of sausage from her pouch on the ground. Then she remained motionless until the dog formed eye contact with her. She marked the correct behaviour using the clicker-like device before again throwing a piece of sausage. Unlike Wallis et al.^27^ and Chapagain et al.^24^, the experimenter did not rustle her pouch when the dog no longer showed interest. The test ended after 15 eye contacts or after 300 s elapsed.

### Sociability tests

#### Greeting test

Before this test, the dog had the opportunity to explore the laboratory room for 2 min in the presence of the owner. At the beginning of the *Greeting* test, the owner stood in the middle of the room and leashed the dog. The experimenter entered the room for the first time, approached the dog-owner pair and said “Hello” to the owner and the dog. She stopped for 1 s in front of the dog, outside the reach of the leash. If the dog approached the experimenter and showed “friendly” or neutral behaviour, she stepped towards the dog, and petted it while continuously speaking to the dog in a friendly tone (Fig. 2B). If the dog showed fearful behaviour, she ignored the dog and talked to the owner for approx. 30 s. After this test the owner unleashed the dog, and the experimenter left the room.

#### Human-directed play test

To measure the playfulness of dogs, we tested them in a situation where they could freely interact with the experimenter. At the beginning of the test, the owner and the dog (off-leash) were in the room, and the experimenter entered with toys (ball and rope) in her hand (Fig. 2C). She offered the toys to the dog, and the dog was free to choose between them. Then, they played with the chosen toy until the first minute elapsed. If the dog was not interested in toys, she tried to initiate social play for 1 min.

### Data collection

We recorded the tests with video cameras, which were connected to computers outside of the testing room. The *Eye contact establishment* test was coded from videos by using Solomon Coder beta 19.08.02 (copyright 2006–2019 by András Péter). The *latency to form each eye contact* with the experimenter was measured from the moment the dog took the food into its mouth until it formed eye contact with the experimenter again. We defined eye contact as the situation in which the dog oriented towards the front of the experimenter and looked up with both eyes into the experimenter's eyes. Eye contact occurrences were indicated by the experimenter with an auditory marker (clicker-like device), thus eye contact was coded from the videos’ audio spectrograms. The videos were coded in 0.1 s time frames. We analysed the first 15 eye contacts of each dog. If a dog went over the allotted 300 s, but formed less than 15 eye contacts, we gave the maximum latency to each remaining trial (e.g. if the dog formed eye contact 13-times within 300 s, in the 14th and 15th trials the latency to form eye contact was set to 300 s and marked as a censored event). In this way, we did not have to exclude those dogs from the analysis which did not form eye contact 15 times within the allotted time period.

The *Greeting* test and *Human-directed play* test were live coded using a binary variable, based on the following definitions: (A) *Greeting* test: (1) *greet immediately in a friendly way* (score = 1; N = 73): the dog approached the experimenter immediately when she entered the room and she could pet it; (2) *no greeting behaviour* (score = 0; N = 52): the dog did not approach the experimenter without calling or she could not pet it; (B) *Human-directed play* test: (1) *high playfulness* (score = 1; N = 60): the dog played enthusiastically with the experimenter, it brought back the ball at least once to her or tugged the rope; (2) *low playfulness* (score = 0; N = 65): the dog did not touch the toys, or it ran after the ball, but did not bring it back to the experimenter, or it took the rope into its mouth a bit, but did not tug it.

A second coder, naïve to the hypotheses of the study, coded a random sample of subjects (~ 20% of dogs). This sample was analysed using intra-class correlations to check the interrater reliability. We found robust reliability for *latency to form eye contact* (ICC: 0.82–1.00, median: 1.00, *p* < 0.001), *greeting behaviour* (ICC: 0.81, *p* < 0.001) and *playfulness with a human* (ICC: 0.91, *p* < 0.001).

### Statistical analysis

We analysed the results using R statistical software (version 3.6.3)^41^ in Rstudio^42^. We used survival analysis, as suggested for latency outcomes in behavioural experiments by Jahn-Eimermacher et al.^43^, as it can handle events which have not occurred within a specified time. We examined each latency per dog (i.e. 15 latencies belong to each dog), thus Mixed Effects Cox Regression Models (“coxme” function of “coxme”^44^ package) were used to analyse the effect of *cephalic index* value, breed function, age and sociability (*greeting behaviour* *score* and *playfulness score*) on the *latency to form eye contact*, with subject ID as a random factor. We also included trial numbers and experimenter identity in the analysis as confounding variables.

Binomial Generalized Linear Models with logit link (“glm” function of “stats”^41^ package) were used to check the possible relationship between the demographic and morphological factors (*cephalic index* value, breed function, age) and the sociability binary scores (*greeting behaviour* and *playfulness with a human*). We also included experimenter identity in the analysis as a confounding variable. To test the independence of the two subtests of sociability, we used a Pearson's Chi-squared test with Yates' continuity correction (“chisq.test” function of “stats”^41^ package).

For the Mixed Effects Cox Regression Models, bottom-up model selection was used (“anova” function of “stats”^41^ package), where the inclusion criteria were a significant likelihood ratio test for each tested variable. The most parsimonious model contained breed function, *playfulness with a human* and trial number as factors, and *cephalic index* value and age as covariates (for more details see Supplementary Table S3). A Tukey post-hoc test was used for comparisons between the three breed function groups (“emmeans” function of “emmeans”^45^ package). For the Binomial Generalized Linear Models, AIC based model selection was applied to find the most parsimonious model using “dredge” function of “MuMIn”^46^ package. According to the model selection, it contained only age as a covariate for both Sociability subtests, and no other factors (for more details see Supplementary Table S4–S5).

We used the “vif” function of the “car”^47^ package to check the possible multicollinearity among the independent variables. The variance inflation factor (VIF) measures how much the variance of any one of the variables is inflated due to multicollinearity in the overall model. If the VIF score is over 5, there is a problem with multicollinearity.

We used the “survfit” function of the “survival”^48^ package and the “ggsurvplot” function of the “survminer”^49^ package to create survival plots and the “ggplot” function of the “ggplot2”^50^ package to produce probability plots.

## Results

All VIF scores were under 1.6, revealing no multicollinearity among the independent variables (for more information, check Supplementary Table S2).

Trial number had a significant effect on *latency to form eye contact*, indicating that dogs learnt about the task during the test (*p* < 0.001). Post-hoc test showed that dogs became faster during the trials, e.g. in the 15th trial, dogs were quicker to establish eye contact with the experimenter than in the 1st trial with a hazard ratio of 2.064 (95% CI = (1.575–2.705), Z = 5.25, *p* < 0.001). We did not find a significant interaction between *cephalic index* and trial number; breed function and trial number; age and trial number or *playfulness with a human* and trial number.

**Figure 3:**
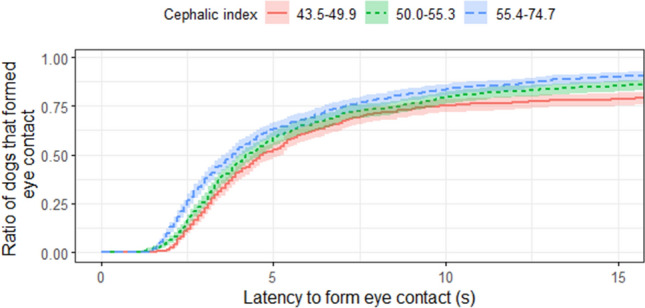
Survival plot for 125 dogs in three head shape groups. *Cephalic index* was a continuous value in the analysis, but the sample was divided into three groups at the 0.33 and 0.67 quartiles for *visualisation purposes only*. On the X axis the *latency to form eye contact* can be read (for visualisation purposes, data are censored at 15 s), while on the Y axis the ratio of dogs that formed eye contact with the experimenter is displayed.

*Cephalic index* had a significant positive association with *latency to form eye contact* with a hazard ratio of 1.055 (95% CI = (1.018–1.094), Z = 2.94, *p* = 0.003; Fig. 3). Dogs with a higher *cephalic index* (shorter headed dogs) formed eye contact faster than dogs with a lower *cephalic index*.

**Figure 4:**
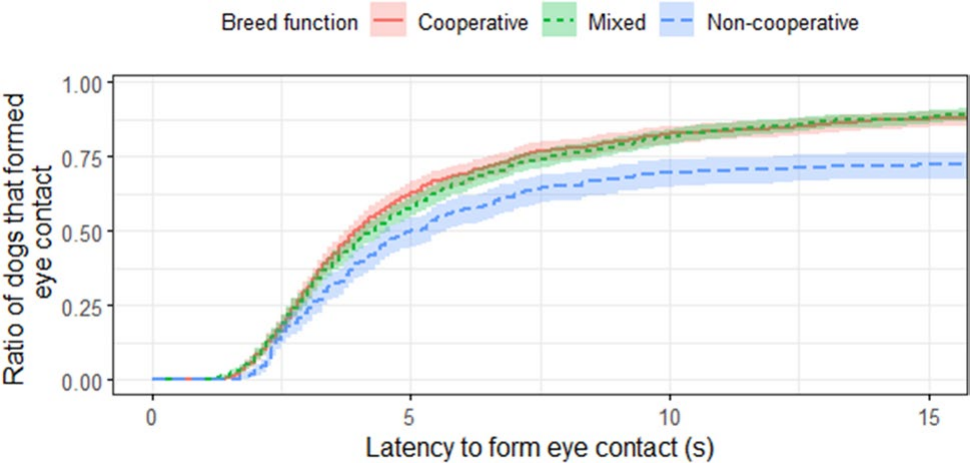
Survival plot for 125 pet dogs in the three breed function groups. E.g. after 15 s elapsed, usually ~ 90% of *cooperative breeds* and *mixed breed dogs* have already formed eye contact with the experimenter, while at the same time only ~ 75% of *non-cooperative breeds* had.

*Breed function* also had a significant effect on *latency to form eye contact* (*p* < 0.001; Fig. 4). A Tukey post-hoc test showed that *cooperative breeds* were more prone to establish eye contact more quickly with the experimenter than *non-cooperative breeds* with a hazard ratio of 2.965 (95% CI = (1.653–5.317), Z = 3.64, *p* < 0.001). *Mixed breed dogs* were also more inclined to form eye contact earlier than *non-cooperative breeds* with a hazard ratio of 2.935 (95%CI = (1.719–5.011), Z = 3.95, *p* < 0.001), while we found no difference between *cooperative breeds* and *mixed breed dogs* (hazard ratio of 1.010 (95% CI = (0.618–1.652), Z = 0.04, *p* = 0.999)).

**Figure 5:**
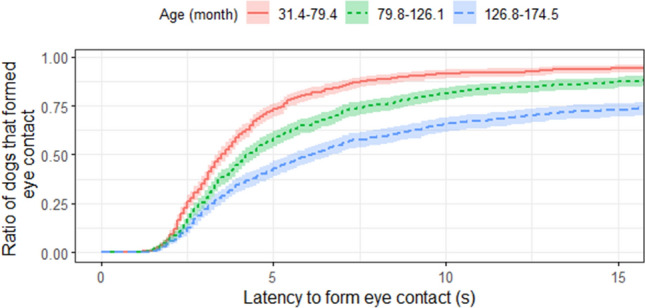
Survival plot for the 125 pet dogs divided into three age groups. Age was a continuous value in the analysis, but the sample was divided into three groups at the 0.33 and 0.67 quartiles for visualisation purposes only. For example, after 10 s elapsed, almost all dogs younger than 6 years (31.4–79.4 months) formed eye contact with the experimenter, while only circa 65% of the dogs older than 10 years (126.8–174.5 months) did so.

**Figure 6:**
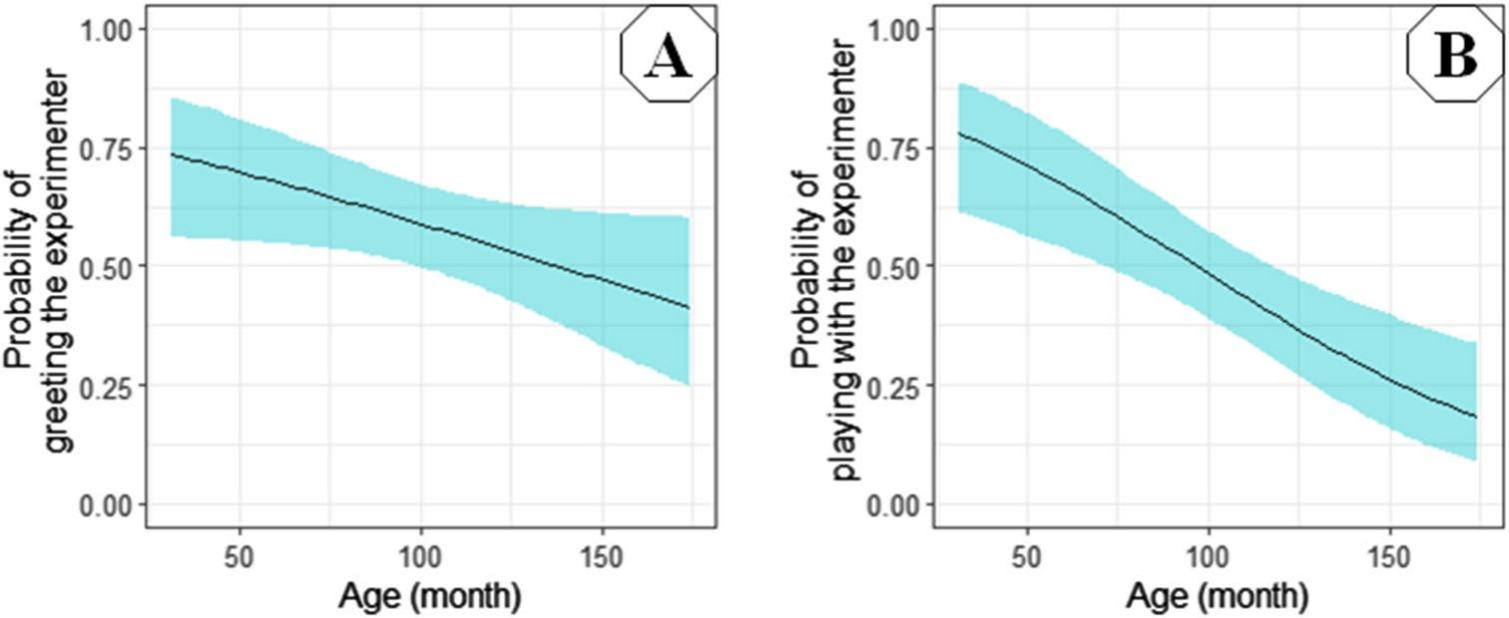
Probability of greeting the experimenter without her needing to call the dog (**A**) and playing more enthusiastically with her (**B**), according to age in months. Younger dogs were more likely to greet the experimenter and had higher playfulness, than the older ones.

Age had a significant negative effect on *latency to form eye contact* with a hazard ratio of 0.986 (95% CI = (0.981–0.991), Z =  − 5.27, *p* < 0.001; Fig. 5), on *greeting an unfamiliar human* with a hazard ratio of 0.991 (95% CI = (0.981–1.000), Z =  − 1.97, *p* = 0.049; Fig. 6A) and on *playfulness with a human* with a hazard ratio of 0.981 (95% CI = (0.971–0.991), Z =  − 3.73, *p* < 0.001; Fig. 6B).

**Figure 7:**
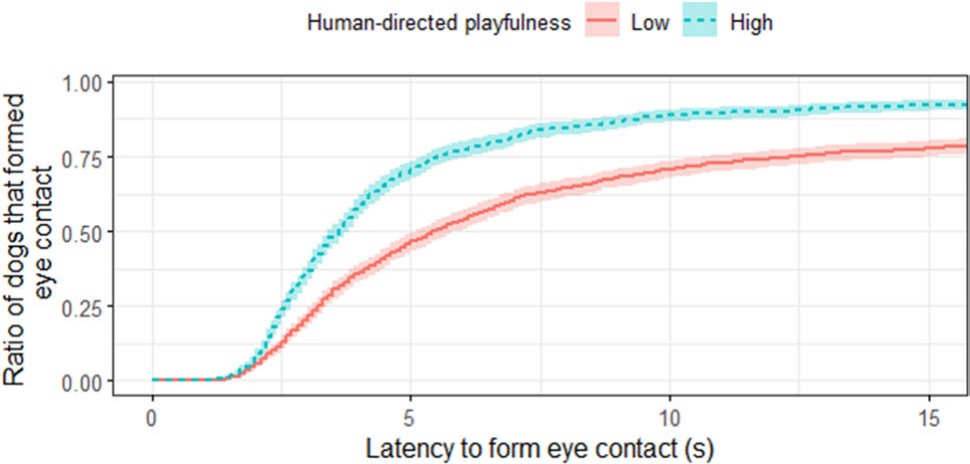
Survival plot for 125 dogs divided into high and low playfulness with a human. E.g. after 10 s elapsed, usually ~ 90% of dogs with *high playfulness* had already formed eye contact with the experimenter, while at the same time only ~ 75% of dogs with *low playfulness* did so.

Finally, among the Sociability tests, only *playfulness with a human* had a significant effect on *latency to form eye contact*. Dogs with *high playfulness* were quicker to establish eye contact with the experimenter than dogs with *low playfulness* with a hazard ratio of 1.673 (95% CI = (1.074–2.608), Z = 2.28, *p* = 0.023; Fig. 7). Dogs’ *greeting behaviour* did not predict their performance in the *Eye contact establishment* test, although the sociability scores (*greeting behaviour* and *playfulness with a human*) were not independent (χ^2^_(1)_ = 14.435, *p* < 0.001). Seventy-three percent of dogs which did not approach the experimenter immediately in the *Greeting* test, also did not play with her in the *Human-directed play* test, while only thirty-seven percent of dogs which approached her without calling, did not play with her.

We found no effect of experimenter identity on any of the three tests, and no effect of *cephalic index* value and breed function on the sociability scores.

## Discussion

**Figure 8:**
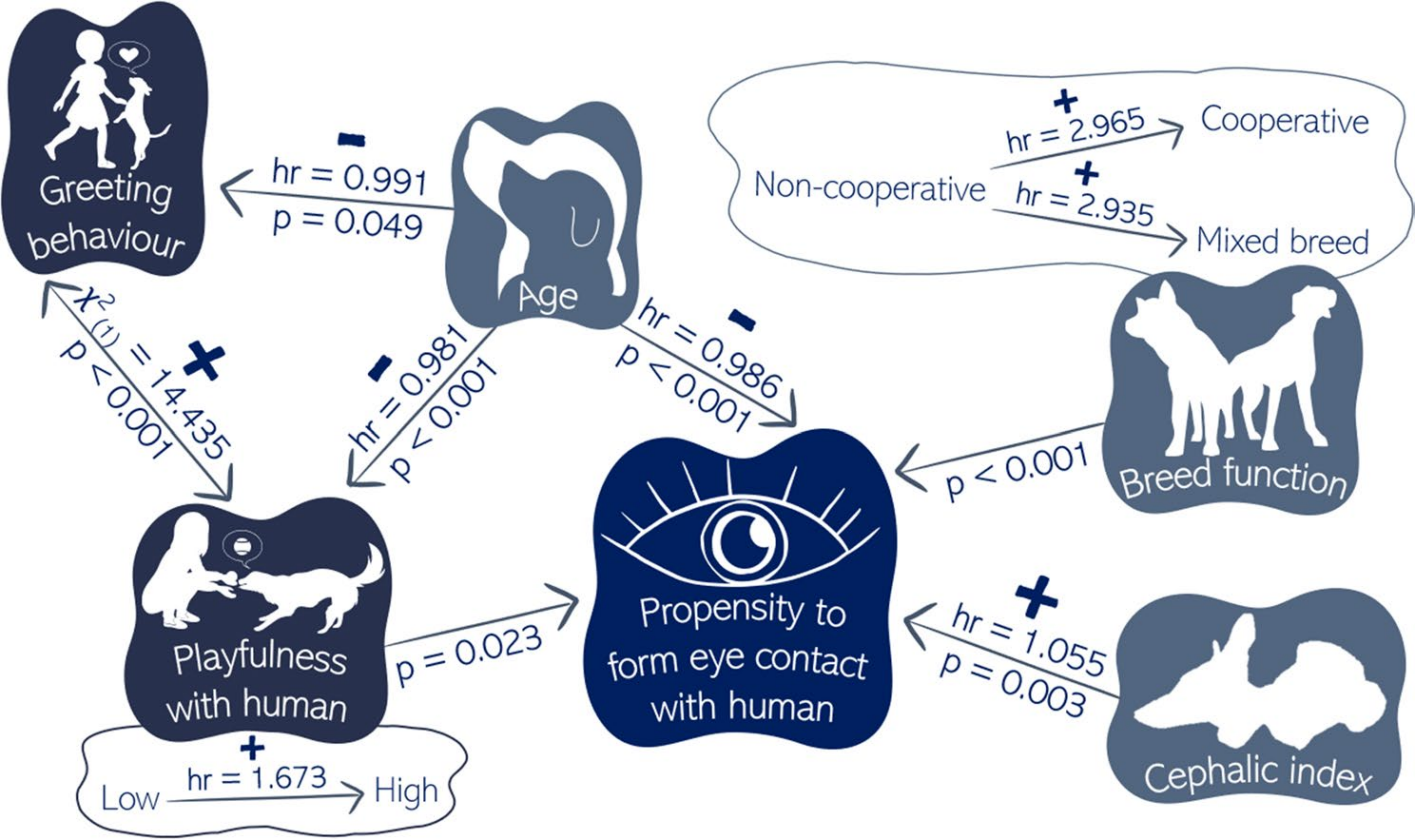
Summary diagram of the main results (created by using drawings designed by Freepik^51^). Each effects’ hazard ratios (hr) and *p* values (*p*) are presented. The levels of categorical variables are presented separately, and the direction of the effects are shown (“ + ” positive and “ − ” negative).

In this study, we examined the effect of four influencing factors *cephalic index*, breed function, age, and sociability on the propensity of pet dogs to form eye contact with a human. Based on the results, there are stable traits that affect dogs’ performance throughout their lifetime, such as head morphology and breed function. In addition, individual characteristics that can change over time, such as age and sociability, also modify the visual communication ability of dogs. The main results are summarized in Fig. 8.

An important finding of this study is the effect of head shape (notably its metric, the *cephalic index*) on dogs’ latency to form eye contact. Dogs with a higher *cephalic index* value (shorter headed dogs) were quicker to make eye contact with the human than dogs with a lower *cephalic index* value (longer headed dogs). This finding is consistent with those of Gácsi et al.^13^ and Bognár et al.^14^ who compared the typical dog head shape groups (see Fig. 1) and found that *brachycephalic* (short-headed) dogs are more successful at following human pointing gestures^13^ and pay more attention to projected faces^14^ than *dolichocephalic* (long-headed) dogs. This may be due to differences in the retinal ganglion cell distribution and thus shorter headed dogs’ better visual acuity^10^. Consequently, shorter headed dogs are more attentive to people, which may make them appear more social and easier to interact with. This might in turn explain the explosion in the popularity of these breeds (such as the Pug^52^ and French bulldog^53^ etc.). The findings of McGreevy et al.^39^ and Stone et al.^16^ are in line with this idea, as they reported that *brachycephalic* dogs are more friendly, interactive and cooperative with unfamiliar humans and stranger-directed fear is most common in *dolichocephalic* dogs. Another factor which can explain the increasing popularity of *brachycephalic* breeds is the “*baby schema effect*”, as mammals have a preference for paedomorphic faces^54–56^. The term “*baby schema*” refers to a set of facial features (i.e. large head and a round face, large eyes etc.), which elicits the so-called “*cute response*”, an increased attention and willingness to care for individuals with infantile features^56^. The characteristics of *brachycephalic* dogs’ heads are in accordance with the baby schema features, thus the owners of these dogs may pay more attention towards them and are more likely to engage in mutual gaze with their animals. Therefore, these dogs may have more opportunity to learn to engage with humans and make eye-contact with them. It is also possible that selection for brachycephaly has been accompanied by selection for seeking eye-contact with humans. Therefore, the underlying causes of the association between the propensity to form eye contact with humans and the head shape of dogs needs to be further tested. It is worth noting that head shape is also linked to brain size, namely that cephalic index negatively correlates with estimated brain weight^57^, which might also influence the behaviour and cognition of these breeds. Further studies regarding the relationship between *cephalic index*, brain size and dog cognition would be desirable. An important question for further research is whether shorter headed dogs' greater visual acuity is correlated with any differences in the brain processes underlying attention.

This study also supports our assumption, that the *cephalic index* scale can be examined individually and as a continuous variable, without the need to form arbitrary head shape groups. Thus, we suggest that further research on the effect of dogs' head shape consider *cephalic index* as a continuous variable and measure it at the individual level, since this could prove more informative.

We also found that breed function affects dogs’ *latency to form eye contact*. As expected, and supporting previous findings^13,18^, *non-cooperative breeds* were slower in the test, and less prone to form eye contact with the experimenter. This suggests that selective breeding for working in visual contact with humans still has a measurable effect on dog behaviour. Surprisingly, there were no differences between *cooperative breeds* and *mixed breed dogs*. We expected that the performance of *mixed breed dogs* would be similar to *non-cooperative breeds*. It is possible that we have tested more *mixed breed dogs* which were mixes of *cooperative breeds* than *non-cooperative breeds*. However, in the current study we could not test this, as the breed makeup of the *mixed breed dogs* in our sample was unknown. Alternatively, as a consequence of domestication, sensitivity to human visual communication was typical in ancient dogs, since early humans likely bred only those individuals which showed more human-like skills^58^, and during modern purebred dogs' intensive selective breeding process—which started 200–300 years ago^59–62^—the *non-cooperative breeds* lost their propensity as they were bred for working independently from the human partner. However, this hypothesis is in conflict with the observation that ancient breeds (i.e. dogs with similar genetic signatures to wolves) were reported to be less prone to form eye contact with humans^19^. In contrast, previous studies suggested that there might be a different, indirect selection force on *mixed breed dogs* compared to purebreds^13,63^, as they mostly live and reproduce on their own, without an owner, but still have to rely on humans for resources. *Mixed breed* dogs living on street with a propensity to form eye contact with humans might have higher fitness, as people may favour such dogs and give them food more readily, which can be crucial to survive on streets. Comparing *mixed breed* dogs with different experience with humans, study found that street dogs did not differ from *mixed breed* pet dogs in the propensity to look at an unfamiliar human^64^. Although we have no information about the life experiences or lineages of the *mixed breed* dogs in our sample, at least 68% of them were adopted from an animal shelter or found on the street. Shelter dogs may have lived for years as pets, but most of the dogs in animal shelters come from the streets. They have therefore adjusted to eating what they find or what they can beg from humans.

Age had a negative effect on performance in all three tests. These results are consistent with other studies in dogs reporting an age-related decrease in dogs’ social attention^27,31^, higher touch sensitivity, and lower companionability and playfulness^65–67^. Our study supports the age-dependent decline in attention and sociability among healthy pet dogs in general, even when considering the dogs’ breed history and head morphology. There are, however, other possible explanations: Older dogs play less, because they may have joint pain or other physical pain which makes playing uncomfortable for them. Owners tend to stop playing with their older dogs^68^, which could also cause decreased playfulness. Older dogs may have a smaller learning ability than younger dogs. Although the differences in learning speed is unlikely to cause the differences found in *latency to form eye contact*, because dogs were able to improve their performance over the test regardless their age. Older dogs may have deficiencies in attentional control and to ignore distracting information (food on the ground or the experimenter's hand, which giving the food), as suggested by previous research^27,31,69^.

From our Sociability tests, only playfulness showed a connection with dogs' propensity to form eye contact with the experimenter. We found that the more playful dogs formed eye contact faster with an unfamiliar human, which is consistent with the findings of Jakovcevic et al.^37^. Their sociability test was performed in the absence of the owner and they differentiated more sociable dogs from less sociable ones by measuring the amount of time the dog spent close to the experimenter. The only measured interactions between the dog and the experimenter were petting and talking. In spite of these differences, our findings are very similar to Jakovcevic et al.'s, suggesting that we observed different expressions of the same relationship in dogs—a positive association between sociability and visual communication with humans.

One major limitation in this study is that we had no data about the animals’ prior experience with the task-relevant behaviour; whether and to what extent the owner trained the dog to form and maintain eye contact with him/her. We only had data on dogs’ previous dog school attendance (yes/no), which had no significant effect on the *latency to form eye contact* with the experimenter in our test. In previous studies, where dogs' full training history was considered, different results were found. They calculated each dog’s training score from a full training history reported by the owner^24,27^. Training score had no significant effect on *latency to form eye contact* with the experimenter in Border collies^27^. When testing in a sample with more breeds, it was found that dogs with a higher training score established eye contact sooner^24^, but the latter study did not consider the dogs' breed function or head shape. To develop a full picture of the connection between head shape, breed function and dogs' propensity to form eye contact with humans, additional studies will be needed that also take into account the dogs' full training history.

This study did not investigate whether differences between individuals in forming eye contact with an unfamiliar person are also predictive of interactions with the owner. Dogs pay more attention to their owner than to unfamiliar individuals^69,70^. But see Kubinyi et al. (submitted) for an opposite result. Familiarity can affect different dog breeds’ attention to humans in varied ways^20^. In Maglieri et al.^20^ German shepherds looked longer at their owner, Czechoslovakian wolfdogs looked longer at the experimenter, while Labrador retrievers paid equal attention to familiar and unfamiliar persons. Kubinyi et al. (submitted) found that dogs can be assigned to four groups based on the frequency of looking at the owner and looking at the experimenter in ambiguous situations. The frequency of looking at the owner and the experimenter correlated (positively) only in one group. Therefore, dogs that are quick to look at an unfamiliar person, may not necessarily respond in the same way to their owner, and vice versa.

In sum, our study suggests that selective breeding for head morphology and different functions impacted dogs’ attention to humans, and consequently their visual communicative abilities. Besides these major effects, individual characteristics, including age and playfulness also influenced the propensity of dogs to form eye contact with an unfamiliar human. We can expect the greatest propensity from short headed dogs, which belong to a visually cooperative breed, that are also young and act playfully with strangers. Since the multicollinearity for the examined predictors was low, the sample groups must be balanced for all of these factors in future studies which investigate dogs’ attention and visual communication abilities.

## Supplementary Information

Supplementary Information 1.

Supplementary Information 2.

Supplementary Information 3.

## Data Availability

All raw data are available as Supplementary material.
