# Supplementary material for: Shorter headed dogs, visually cooperative breeds, younger and playful dogs form eye contact faster with an unfamiliar human
Source: Sci Rep. 2021 Apr 29;11:9293. doi: 10.1038/s41598-021-88702-w (PMC8084920; doi:10.1038/s41598-021-88702-w)
Supplement: Supplementary file 1 — Supplementary Information 1. [file 41598_2021_88702_MOESM1_ESM.pdf]

Shorter headed dogs, visually cooperative breeds, younger and playful dogs form eye contact faster with an unfamiliar human

Zsófia Bognár[1*], Dóra Szabó[1], Alexandra Deés[1], Enikő Kubinyi[1]

[1] Senior Family Dog Project, Department of Ethology, Eötvös Loránd University, Budapest, Hungary

*Corresponding author: bognarzsof@gmail.com

Supplementary information

Supplementary Table S1. Name, breed, breed function, *cephalic index* value, sex (and sexual status), age of the dogs and the experimenter's monogram, who conducted the test.

	Dog	Breed	Cephalic index	Breed function	Age (month)	Sex (i=intact)	Experimenter
**1**	Áfonya1	Mixed	51.54	Mixed	138.8	Female	ZB
**2**	Áfonya2	Mixed	48.40	Mixed	51.2	Female	VH
**3**	Alec	Labrador Retriever	53.89	Cooperative	126.1	Male (i)	ZB
**4**	Alfi	Belgian Shepherd Dog (Tervuren)	48.41	Cooperative	55.7	Male	AD
**5**	Alice	Mixed	56.79	Mixed	163.4	Female	ZB
**6**	Andi	Mixed	55.18	Mixed	79.3	Female (i)	BS
**7**	Angelo	Rhodesian Ridgeback	48.38	Non-cooperative	104.5	Male	DS
**8**	Apache	Border Collie	57.66	Cooperative	137.9	Male (i)	DS
**9**	Archibald	Mixed	47.71	Mixed	167.4	Male (i)	ZB
**10**	Bajszi	Mixed	44.93	Mixed	131.3	Female (i)	RB
**11**	Balu	Mixed	53.79	Mixed	73.3	Male	AD
**12**	Bambi	Mixed	53.17	Mixed	96.9	Female	AE
**13**	Barbason	Whippet	48.91	Non-cooperative	110.4	Male	DS
**14**	Barka	English Cocker Spaniel	45.66	Cooperative	76.9	Male (i)	AE
**15**	Bejgli	Beagle	58.79	Non-cooperative	45.3	Male	BS
**16**	Bijou	Whippet	48.18	Non-cooperative	142.8	Female	ZB
**17**	Bizsu	Labrador Retriever	56.16	Cooperative	98.7	Female	RB
**18**	Bob	Mixed	61.50	Mixed	43.8	Male	ZB
**19**	Bodor	Mixed	54.31	Mixed	140.5	Male	SM
**20**	Bodza1	Giant Schnauzer	47.45	Non-cooperative	113.6	Male	SM
**21**	Bodza2	Golden Retriever	57.56	Cooperative	115.0	Male (i)	VH
**22**	Bogyó1	Mixed	56.14	Mixed	135.4	Male	ZB
**23**	Bogyó2	Beagle	61.47	Non-cooperative	75.1	Male (i)	AE
**24**	Borisz	Whippet	48.42	Non-cooperative	130.2	Male (i)	BS
**25**	Brutus	Mixed	59.25	Mixed	134.7	Male	DS
**26**	Bucka	Mixed	49.24	Mixed	124.7	Female	SM
**27**	Buddha	Bouvier des Flandres	46.00	Cooperative	36.4	Male (i)	AD
**28**	Bukó	Mixed	55.92	Mixed	101.7	Male	VH
**29**	Creasy	Bernese Mountain Dog	52.19	Non-cooperative	139.9	Male	DS
**30**	Csingi	Mixed	56.66	Mixed	106.5	Female	SM
**31**	Csoki	German Shorthaired Pointer	49.51	Cooperative	132.5	Female (i)	SM
**32**	Csutka	Mixed	48.81	Mixed	59.1	Female	ZB
**33**	Cuki	Mixed	49.77	Mixed	80.8	Female	ZB
**34**	Dancey	Mixed	58.70	Mixed	75.9	Female (i)	BS
**35**	Dása	Mixed	52.58	Mixed	94.9	Female	DS
**36**	Delko	Doberman Pinscher	43.47	Non-cooperative	58.1	Male	BS
**37**	Dexter	Mixed	54.11	Mixed	98.7	Male	SM
**38**	Dolli	Boxer	67.47	Non-cooperative	43.2	Female	AD
**39**	Dolores	Mixed	54.36	Mixed	42.5	Female	BS
**40**	Dominó	Border Collie	55.11	Cooperative	128.6	Female	RB
**41**	Dorell	Dalmatian	49.54	Non-cooperative	43.8	Male	BS
**42**	Döme	English Cocker Spaniel	47.53	Cooperative	59.8	Male	AD
**43**	Elza	German Wirehaired Pointer	48.92	Cooperative	138.0	Female	ZB
**44**	Fickó	Mixed	43.75	Mixed	67.1	Male	VH
**45**	Frida	Basset Hound	46.28	Non-cooperative	37.3	Female	AD
**46**	Frutti	Mixed	52.23	Mixed	95.7	Female	SM
**47**	Füge	Mixed	52.71	Mixed	75.7	Female	AE
**48**	Füles	Mixed	48.38	Mixed	174.5	Female	BS
**49**	Füli	Mixed	51.47	Mixed	163.6	Male	DS
**50**	Fülöp	Mixed	53.17	Mixed	123.0	Male	ZB
**51**	Gonzó	Siberian Husky	54.85	Non-cooperative	130.1	Male	BS
**52**	Hetty	Cavalier King Charles Spaniel	67.58	Cooperative	126.8	Female	ZB
**53**	Huba	Mixed	48.34	Mixed	76.5	Male (i)	ZB
**54**	Jackson	Doberman Pinscher	45.19	Non-cooperative	63.3	Male (i)	VH
**55**	Janda	Mixed	58.16	Mixed	137.1	Male	DS
**56**	Jenna	American Pit Bull Terrier	56.72	Non-cooperative	53.6	Female	BS
**57**	Jollie	Vizsla	55.33	Cooperative	79.4	Female	ZB
**58**	Kai	Mixed	50.77	Mixed	37.6	Male (i)	AD
**59**	Káldor	Mixed	50.45	Mixed	106.2	Male (i)	DS
**60**	Kiki	Border Collie	55.57	Cooperative	36.9	Female (i)	ZB
**61**	Knut	Mixed	67.99	Mixed	134.8	Male	DS
**62**	Koda	Siberian Husky	54.49	Non-cooperative	40.3	Male (i)	ZB
**63**	Kópé1	Mixed	52.10	Mixed	147.9	Male	VH
**64**	Kópé2	Cairn Terrier	66.39	Non-cooperative	122.9	Male	SM

**65**	Kópé3	Transylvanian Hound	49.44	Non-cooperative	62.3	Male	BS
**66**	Kuszi	Mixed	52.51	Mixed	143.1	Female	BS
**67**	Lajka	Mixed	49.85	Mixed	35.8	Female	AD
**68**	Lara	Border Collie	56.80	Cooperative	33.5	Female	ZB
**69**	Letty	Mixed	48.32	Mixed	57.7	Female	AD
**70**	Lisa	Vizsla	50.73	Cooperative	162.6	Female	ZB
**71**	Liza	Vizsla	50.50	Cooperative	163.4	Female	DS
**72**	Lizi	Border Collie	60.26	Cooperative	106.5	Female	BS
**73**	Luna	Mixed	57.50	Mixed	98.7	Female	VH
**74**	Lupita	Border Collie	54.52	Cooperative	122.6	Female	DS
**75**	Maci	Mixed	58.07	Mixed	104.0	Male	ZB
**76**	Maja	Mixed	49.43	Mixed	127.6	Female	RB
**77**	Manó	Mixed	52.74	Mixed	96.2	Female	VH
**78**	Mara	Shetland Sheepdog	45.01	Cooperative	40.5	Female	ZB
**79**	Mazsi	Mixed	53.70	Mixed	75.6	Female	ZB
**80**	Merlin	Labrador Retriever	56.27	Cooperative	97.0	Male	ZB
**81**	Metró	Mixed	51.27	Mixed	109.1	Male	DS
**82**	Mira	Border Collie	57.69	Cooperative	144.1	Female	BS
**83**	Mixi	Mixed	53.86	Mixed	39.1	Female	VH
**84**	Molly	English Cocker Spaniel	44.75	Cooperative	58.9	Female	AD
**85**	Momo	American Pit Bull Terrier	60.51	Non-cooperative	37.8	Male (i)	AD
**86**	Monti	Boxer	74.68	Non-cooperative	108.2	Male	DS
**87**	Móric	Border Collie	54.32	Cooperative	150.8	Male	BS
**88**	Néró	Labrador Retriever	50.82	Cooperative	143.0	Male (i)	BS
**89**	Niké	Border Collie	57.76	Cooperative	152.3	Female	BS
**90**	Nina	Mixed	57.43	Mixed	43.9	Female (i)	AE
**91**	Nova	Mixed	44.83	Mixed	39.4	Male (i)	ZB
**92**	Olivér	Mixed	69.84	Mixed	98.9	Male	DS
**93**	Panka1	Mixed	48.81	Mixed	147.3	Female	BS
**94**	Panka2	Vizsla	50.02	Cooperative	122.5	Female	ZB
**95**	Pedro	Poodle	50.66	Cooperative	79.7	Male (i)	BS
**96**	Pepi	Mixed	56.73	Mixed	160.9	Male (i)	RB
**97**	Perec	Border Collie	49.16	Cooperative	31.4	Male	AE
**98**	Pinky	Cairn Terrier	70.84	Non-cooperative	105.3	Female	ZB
**99**	Pletyka	Mudi	59.70	Cooperative	138.7	Female	ZB
**100**	Queeny	Border Collie	55.35	Cooperative	123.2	Female	BS
**101**	Roger	Border Collie	51.22	Cooperative	137.2	Male	DS
**102**	Roni	Shetland Sheepdog	44.49	Cooperative	93.4	Male	SM
**103**	Rozi1	Border Collie	58.12	Cooperative	132.1	Female	BS
**104**	Rozi2	Mixed	55.22	Mixed	100.4	Female	SM
**105**	Rudi	Vizsla	55.32	Cooperative	127.9	Male	BS
**106**	Sunny	Mixed	53.52	Mixed	36.5	Female	ZB
**107**	Szamóca	Whippet	47.10	Non-cooperative	127.3	Female	RB
**108**	Tappancs	Mixed	62.61	Mixed	120.4	Male	DS
**109**	Tapsi	Whippet	47.39	Non-cooperative	119.7	Male	ZB
**110**	Tashi	Tibetan Terrier	58.83	Cooperative	111.2	Male	ZB
**111**	Tasli	Mixed	53.75	Mixed	122.6	Female	ZB
**112**	Teo	German Shepherd Dog	49.04	Cooperative	131.7	Male	BS
**113**	Terka	Wirehaired Vizsla	45.16	Cooperative	69.7	Female (i)	ZB
**114**	Titi	Tibetan Terrier	61.42	Cooperative	137.3	Male (i)	RB
**115**	Tony	Beagle	55.32	Non-cooperative	96.3	Male (i)	DS
**116**	Vackor	Puli	57.74	Cooperative	128.6	Male	BS
**117**	Velvet	Whippet	49.73	Non-cooperative	142.1	Male	VH
**118**	Vilja	Airedale Terrier	52.11	Non-cooperative	113.2	Female	DS
**119**	Viola	Mixed	54.55	Mixed	41.8	Female	ZB
**120**	Zazi	Mixed	62.29	Mixed	150.1	Female (i)	SM
**121**	Zeller	Airedale Terrier	48.28	Non-cooperative	43.8	Female (i)	VH
**122**	Zenit	Shetland Sheepdog	56.18	Cooperative	122.1	Female (i)	DS
**123**	Zserbó1	Mixed	53.56	Mixed	108.4	Male	ZB
**124**	Zserbó2	Vizsla	50.83	Cooperative	111.6	Male	AE
**125**	Zsömi	Mixed	47.15	Mixed	135.2	Female	RB

Supplementary Table S2. VIF scores of the independent variables.

Independent variables	VIF score	Df
Cephalic index	1.016	1
Breed function group	1.061	2
Age	1.421	1
Greeting behaviour	1.269	1
Playfulness	1.547	1
Trial number	1.008	14

Details of model selection

We also added the full script of the analysis (Bognar_et_al_EyeContact_ms_suppl_analysis.pdf) as supplementary material.

Supplementary Table S3. Results of Mixed Effects Cox Regression Models' bottom-up model selection.

Models	loglik	Chisq	Df	P(>|Chi|)
Nullmodel	-10920			
Nullmodel+ Cephalic index	-10918	3.005	1	0.083
Nullmodel + Breed group	-10918	3.729	2	0.155
Nullmodel + Age	-10915	9.516	1	0.002
Nullmodel + Greeting	-10921	2.411	1	0.121
Nullmodel + Playfulness	-10920	1.375	1	0.241
**Nullmodel + Trial number**	**-10866**	**108.23**	**14**	**< 0.001**
Nullmodel + Trial number + Cephalic index	-10864	3.223	1	0.073
**Nullmodel + Trial number + Breed group**	**-10861**	**9.753**	**2**	**0.008**
Nullmodel + Trial number + Age	-10864	3.676	1	0.055
Nullmodel + Trial number + Greeting	-10866	1.294	1	0.255
Nullmodel + Trial number + Playfulness	-10870	8.369	1	0.004
Nullmodel + Trial number + Breed group + Cephalic index	-10864	6.449	1	0.011
**Nullmodel + Trial number + Breed group + Age**	**-10856**	**9.963**	**1**	**0.002**
Nullmodel + Trial number + Breed group + Greeting	-10864	6.870	1	0.009
Nullmodel + Trial number + Breed group + Playfulness	-10865	7.973	1	0.005
**Nullmodel + Trial number + Breed group + Age + Cephalic index**	**-10851**	**10.066**	**1**	**0.002**
Nullmodel + Trial number + Breed group + Age + Greeting	-10858	3.757	1	0.053
Nullmodel + Trial number + Breed group + Age + Playfulness	-10858	3.645	1	0.056
Nullmodel + Trial number + Breed group + Age + Cephalic index + Greeting	-10852	2.780	1	0.095
**Nullmodel + Trial number + Breed group + Age + Cephalic index + Playfulness**	**-10852**	**2.883**	**1**	**0.090**
Nullmodel + Trial number + Breed group + Age + Cephalic index + Playfulness + Greeting	-10853	2.214	1	0.137

Although at the stage of the bottom-up model selection when we added Playfulness to the model, the statistic indicated a trend for worsening our model, in the end we decided to add it anyway because the difference is not significant, but Playfulness has a significant impact on our examined variable. Unlike Greeting behaviour, which had no effect.

Supplementary Table S4. Results of Binomial Generalized Linear Models' AIC based model selection for Greeting behaviour (with "dredge" function)

Intercept	Cephalic index	Breed group	Age	df	loglik	AICc	delta	weight
**1.313**			**-0.010**	**2**	**-82.864**	**169.8**	**0.00**	**0.263**
2.032		+	-0.011	4	-80.787	169.9	0.08	0.252
-0.065	0.027		-0.010	3	-82.507	171.2	1.39	0.131
0.528	0.029	+	-0.012	5	-80.367	171.2	1.41	0.130
0.339				1	-84.871	171.8	1.95	0.099
0.802		+		3	-83.315	172.8	3.00	0.059
-0.576	0.017			2	-84.717	173.5	3.71	0.041
-0.200	0.019	+		4	-83.131	174.6	4.77	0.024

Supplementary Table S5. Results of Binomial Generalized Linear Models' AIC based model selection for Playfulness (with "dredge" function)

Intercept	Cephalic index	Breed group	Age	df	loglik	AICc	delta	weight
**1.867**			**-0.019**	**2**	**-78.693**	**161.5**	**0.00**	**0.488**
2.508		+	-0.021	4	-77.229	162.8	1.31	0.254
1.768	0.002		-0.019	3	-78.691	163.6	2.10	0.171
2.274	0.005	+	-0.021	5	-77.219	164.9	3.46	0.087
-0.080				1	-86.543	175.1	13.64	0.001
0.739	-0.015			2	-86.416	176.9	15.45	0.000
0.191		+		3	-85.861	177.9	16.44	0.000
0.974	-0.015	+		4	-85.745	179.8	18.34	0.000
