# Supplementary material for: Shorter headed dogs, visually cooperative breeds, younger and playful dogs form eye contact faster with an unfamiliar human
Source: Sci Rep. 2021 Apr 29;11:9293. doi: 10.1038/s41598-021-88702-w (PMC8084920; doi:10.1038/s41598-021-88702-w)
Supplement: Supplementary file 2 — Supplementary Information 2. [file 41598_2021_88702_MOESM2_ESM.pdf]

Shorter headed dogs, visually cooperative breeds, younger and playful dogs form eye contact faster with an unfamiliar human

Zsófia Bognár[1*], Dóra Szabó[1], Alexandra Deés[1], Enikő Kubinyi[1]

[1] Senior Family Dog Project, Department of Ethology, Eötvös Loránd University, Budapest, Hungary

*Corresponding author: bognarzsof@gmail.com

1. Starting the analysis

a. Import data

```
library(readxl)

EC_data <- read_excel("D:/Where/Is/The file/On your computer/Bognar_et_al_EyeContact_ms_sup
pl_data.xlsx", sheet = "Data")

EC_data$DogID <- factor(EC_data$DogID)
EC_data$CI_fig <- factor(EC_data$CI_fig, levels = c('43.5-49.9', '50.0-55.3','55.4-74.7'))
EC_data$BreedGroup <- factor(EC_data$BreedGroup)
EC_data$Age_fig <- factor(EC_data$Age_fig,levels = c('31.4-79.4','79.8-126.1','126.8-174.5'
))
EC_data$Greet <- factor(EC_data$Greet)
EC_data$Play <- factor(EC_data$Play)
EC_data$Sex <- factor(EC_data$Sex)
EC_data$Neutered <- factor(EC_data$Neutered)
EC_data$DogSchool <- factor(EC_data$DogSchool)
EC_data$Exp <- factor(EC_data$Exp)
EC_data$Trial <- factor(EC_data$Trial,levels = c('1','2','3','4','5','6','7','8','9','10','
11','12','13','14','15'))
EC_data$Event <- as.numeric(EC_data$Event)

Soc_data <- subset(EC_data, Trial==1) # contains only one data point for each dog; for demo
graphy and sociability analysis

Soc_data_fig <- read_excel("D:/ Where/Is/The file/On your computer/Bognar_et_al_EyeContact_
ms_suppl_data.xlsx", sheet = "Data")
Soc_data_fig <- subset(Soc_data_fig, Trial==1) # separate datasheet is needed for sociabili
ty tests' figures

options(digits=4) # show only 4 digits
```

b. Data verification

```
library(emmeans)

#Possible correlations
BreedCI_mod <- lm(CI ~ BreedGroup, Soc_data) # CI of breed groups
pairs(emmeans(BreedCI_mod, ~BreedGroup))

##  contrast        estimate   SE  df t.ratio p.value
##  Coop - Mixed      -0.295 1.22 122 -0.241  0.9685
##  Coop - Noncoop    -0.561 1.48 122 -0.380  0.9235
##  Mixed - Noncoop   -0.267 1.40 122 -0.190  0.9803
##
## P value adjustment: tukey method for comparing a family of 3 estimates

summary(BreedCI_mod)

##
## Call:
## lm(formula = CI ~ BreedGroup, data = Soc_data)
##
## Residuals:
```

```
##     Min      1Q Median      3Q     Max 
## -10.37  -4.36  -0.40   3.16  20.84 
## 
## Coefficients:
##                     Estimate Std. Error t value Pr(>|t|)    
## (Intercept)           53.275      0.924   57.65   <2e-16 ***
## BreedGroupMixed        0.295      1.223    0.24     0.81    
## BreedGroupNoncoop      0.561      1.477    0.38     0.70    
## ---
## Signif. codes:  0 '***' 0.001 '**' 0.01 '*' 0.05 '.' 0.1 ' ' 1
## 
## Residual standard error: 5.99 on 122 degrees of freedom
## Multiple R-squared:  0.00122,    Adjusted R-squared:  -0.0152 
## F-statistic: 0.0746 on 2 and 122 DF,  p-value: 0.928
```

```
BreedAge_mod <- lm(Age ~ BreedGroup, Soc_data) # Age of breed groups
pairs(emmeans(BreedAge_mod, ~BreedGroup))
```

```
##  contrast        estimate   SE  df t.ratio p.value
##  Coop - Mixed        6.37 7.93 122 0.802   0.7022 
##  Coop - Noncoop     18.39 9.59 122 1.919   0.1377 
##  Mixed - Noncoop    12.03 9.11 122 1.321   0.3863 
## 
## P value adjustment: tukey method for comparing a family of 3 estimates
```

```
summary(BreedAge_mod)
```

```
## 
## Call:
## lm(formula = Age ~ BreedGroup, data = Soc_data)
## 
## Residuals:
##     Min      1Q Median      3Q     Max 
## -76.30 -30.74   6.99  30.30  73.16 
## 
## Coefficients:
##                     Estimate Std. Error t value Pr(>|t|)    
## (Intercept)           107.67       6.00   17.95   <2e-16 ***
## BreedGroupMixed        -6.37       7.93   -0.80    0.424    
## BreedGroupNoncoop     -18.39       9.59   -1.92    0.057 .  
## ---
## Signif. codes:  0 '***' 0.001 '**' 0.01 '*' 0.05 '.' 0.1 ' ' 1
## 
## Residual standard error: 38.9 on 122 degrees of freedom
## Multiple R-squared:  0.0294, Adjusted R-squared:  0.0135 
## F-statistic: 1.85 on 2 and 122 DF,  p-value: 0.162
```

```
cor(Soc_data$Age, Soc_data$CI) #Age and CI
```

```
## [1] 0.1365
```

```
cor.test(Soc_data$Age, Soc_data$CI)
```

```
## 
##  Pearson's product-moment correlation
## 
## data:  Soc_data$Age and Soc_data$CI
## t = 1.5, df = 123, p-value = 0.1
```

```
## alternative hypothesis: true correlation is not equal to 0
## 95 percent confidence interval:
##  -0.04012  0.30476
## sample estimates:
##    cor
## 0.1365

#Calculating means and SEs for each breed group
Soc_data_Coop <- subset(Soc_data, BreedGroup=="Coop")
mean(Soc_data_Coop$CI)

## [1] 53.28

sd(Soc_data_Coop$CI)

## [1] 5.279

mean(Soc_data_Coop$Age)

## [1] 107.7

sd(Soc_data_Coop$Age)

## [1] 38.1

Soc_data_Noncoop <- subset(Soc_data, BreedGroup=="Noncoop")
mean(Soc_data_Noncoop$CI)

## [1] 53.84

sd(Soc_data_Noncoop$CI)

## [1] 8.274

mean(Soc_data_Noncoop$Age)

## [1] 89.27

sd(Soc_data_Noncoop$Age)

## [1] 37.9

Soc_data_Mixed <- subset(Soc_data, BreedGroup=="Mixed")
mean(Soc_data_Mixed$CI)

## [1] 53.57

sd(Soc_data_Mixed$CI)

## [1] 5.141

mean(Soc_data_Mixed$Age)

## [1] 101.3

sd(Soc_data_Mixed$Age)

## [1] 39.87

#Sociability scores
tbl <- table(Soc_data$Greet, Soc_data$Play)
chisq.test(tbl)
```

```
##
##  Pearson's Chi-squared test with Yates' continuity correction
##
## data:  tbl
## X-squared = 14, df = 1, p-value = 1e-04
```

```
#Possible multicollinearity
library(coxme)

library(car)

EC_mod <- coxme(Surv(LEC, Event) ~ CI + BreedGroup + Age + Greet + Play + Trial + (1|DogID)
, data = EC_data)

car::vif(EC_mod) # Calculating VIF scores

##              GVIF Df GVIF^(1/(2*Df))
## CI          1.016  1           1.008
## BreedGroup 1.061  2           1.015
## Age         1.421  1           1.192
## Greet       1.269  1           1.127
## Play        1.547  1           1.244
## Trial       1.008 14           1.000

# If a VIF is greater than 10, you have high multicollinearity and the variation will seem
larger and the factor will appear to be more influential
#than it is. If VIF is closer to 1, then the model is much stronger, as the factors are not
impacted by correlation with other factors.
```

2. Model selections

a. Eye contact establishment test

```
EC_mod0 <- coxme(Surv(LEC, Event) ~ 1 + (1|DogID), data = EC_data) # null modell

#First step
CI_mod <- update(EC_mod0, .~. + CI)
anova(EC_mod0, CI_mod)

## Analysis of Deviance Table
##  Cox model: response is  Surv(LEC, Event)
##  Model 1: ~1 + (1 | DogID)
##  Model 2: ~(1 | DogID) + CI
##   loglik Chisq Df P(>|Chi|)
## 1 -10920
## 2 -10918     3  1     0.083 .
## ---
## Signif. codes:  0 '***' 0.001 '**' 0.01 '*' 0.05 '.' 0.1 ' ' 1

Breed_mod <- update(EC_mod0, .~. + BreedGroup)
anova(EC_mod0, Breed_mod)

## Analysis of Deviance Table
##  Cox model: response is  Surv(LEC, Event)
##  Model 1: ~1 + (1 | DogID)
##  Model 2: ~(1 | DogID) + BreedGroup
##   loglik Chisq Df P(>|Chi|)
```

```
## 1 -10920
## 2 -10918  3.73  2      0.15
```

```
Age_mod <- update(EC_mod0, .~. + Age)
anova(EC_mod0, Age_mod)
```

```
## Analysis of Deviance Table
##  Cox model: response is  Surv(LEC, Event)
##  Model 1: ~1 + (1 | DogID)
##  Model 2: ~(1 | DogID) + Age
##   loglik Chisq Df P(>|Chi|)
## 1 -10920
## 2 -10915  9.52  1     0.002 **
## ---
## Signif. codes:  0 '***' 0.001 '**' 0.01 '*' 0.05 '.' 0.1 ' ' 1
```

```
Greet_mod <- update(EC_mod0, .~. + Greet)
anova(EC_mod0, Greet_mod)
```

```
## Analysis of Deviance Table
##  Cox model: response is  Surv(LEC, Event)
##  Model 1: ~1 + (1 | DogID)
##  Model 2: ~(1 | DogID) + Greet
##   loglik Chisq Df P(>|Chi|)
## 1 -10920
## 2 -10921  2.41  1      0.12
```

```
Play_mod <- update(EC_mod0, .~. + Play)
anova(EC_mod0, Play_mod)
```

```
## Analysis of Deviance Table
##  Cox model: response is  Surv(LEC, Event)
##  Model 1: ~1 + (1 | DogID)
##  Model 2: ~(1 | DogID) + Play
##   loglik Chisq Df P(>|Chi|)
## 1 -10920
## 2 -10920  1.37  1      0.24
```

```
Trial_mod <- update(EC_mod0, .~. + Trial)
anova(EC_mod0, Trial_mod) #*
```

```
## Analysis of Deviance Table
##  Cox model: response is  Surv(LEC, Event)
##  Model 1: ~1 + (1 | DogID)
##  Model 2: ~(1 | DogID) + Trial
##   loglik Chisq Df P(>|Chi|)
## 1 -10920
## 2 -10866   108 14    <2e-16 ***
## ---
## Signif. codes:  0 '***' 0.001 '**' 0.01 '*' 0.05 '.' 0.1 ' ' 1
```

```
#Second step
CI_mod <- update(Trial_mod, .~. + CI)
anova(Trial_mod, CI_mod)
```

```
## Analysis of Deviance Table
##  Cox model: response is  Surv(LEC, Event)
##  Model 1: ~(1 | DogID) + Trial
##  Model 2: ~(1 | DogID) + Trial + CI
```

```
##   loglik Chisq Df P(>|Chi|)
## 1 -10866
## 2 -10864  3.22  1     0.073 .
## ---
## Signif. codes:  0 '***' 0.001 '**' 0.01 '*' 0.05 '.' 0.1 ' ' 1

Breed_mod <- update(Trial_mod, .~. + BreedGroup)
anova(Trial_mod, Breed_mod) #*

## Analysis of Deviance Table
##  Cox model: response is  Surv(LEC, Event)
##  Model 1: ~(1 | DogID) + Trial
##  Model 2: ~(1 | DogID) + Trial + BreedGroup
##   loglik Chisq Df P(>|Chi|)
## 1 -10866
## 2 -10861  9.75  2    0.0076 **
## ---
## Signif. codes:  0 '***' 0.001 '**' 0.01 '*' 0.05 '.' 0.1 ' ' 1

Age_mod <- update(Trial_mod, .~. + Age)
anova(Trial_mod, Age_mod)

## Analysis of Deviance Table
##  Cox model: response is  Surv(LEC, Event)
##  Model 1: ~(1 | DogID) + Trial
##  Model 2: ~(1 | DogID) + Trial + Age
##   loglik Chisq Df P(>|Chi|)
## 1 -10866
## 2 -10864  3.68  1     0.055 .
## ---
## Signif. codes:  0 '***' 0.001 '**' 0.01 '*' 0.05 '.' 0.1 ' ' 1

Greet_mod <- update(Trial_mod, .~. + Greet)
anova(Trial_mod, Greet_mod)

## Analysis of Deviance Table
##  Cox model: response is  Surv(LEC, Event)
##  Model 1: ~(1 | DogID) + Trial
##  Model 2: ~(1 | DogID) + Trial + Greet
##   loglik Chisq Df P(>|Chi|)
## 1 -10866
## 2 -10866  1.29  1      0.26

Play_mod <- update(Trial_mod, .~. + Play)
anova(Trial_mod, Play_mod)

## Analysis of Deviance Table
##  Cox model: response is  Surv(LEC, Event)
##  Model 1: ~(1 | DogID) + Trial
##  Model 2: ~(1 | DogID) + Trial + Play
##   loglik Chisq Df P(>|Chi|)
## 1 -10866
## 2 -10870  8.37  1    0.0038 **
## ---
## Signif. codes:  0 '***' 0.001 '**' 0.01 '*' 0.05 '.' 0.1 ' ' 1

#Third step
CI_mod <- update(Breed_mod, .~. + CI)
anova(Breed_mod, CI_mod)
```

```
## Analysis of Deviance Table
##  Cox model: response is  Surv(LEC, Event)
##  Model 1: ~(1 | DogID) + Trial + BreedGroup
##  Model 2: ~(1 | DogID) + Trial + BreedGroup + CI
##   loglik Chisq Df P(>|Chi|)
## 1 -10861
## 2 -10864  6.45  1     0.011 *
## ---
## Signif. codes:  0 '***' 0.001 '**' 0.01 '*' 0.05 '.' 0.1 ' ' 1

Age_mod <- update(Breed_mod, .~. + Age)
anova(Breed_mod, Age_mod) #*

## Analysis of Deviance Table
##  Cox model: response is  Surv(LEC, Event)
##  Model 1: ~(1 | DogID) + Trial + BreedGroup
##  Model 2: ~(1 | DogID) + Trial + BreedGroup + Age
##   loglik Chisq Df P(>|Chi|)
## 1 -10861
## 2 -10856  9.96  1    0.0016 **
## ---
## Signif. codes:  0 '***' 0.001 '**' 0.01 '*' 0.05 '.' 0.1 ' ' 1

Greet_mod <- update(Breed_mod, .~. + Greet)
anova(Breed_mod, Greet_mod)

## Analysis of Deviance Table
##  Cox model: response is  Surv(LEC, Event)
##  Model 1: ~(1 | DogID) + Trial + BreedGroup
##  Model 2: ~(1 | DogID) + Trial + BreedGroup + Greet
##   loglik Chisq Df P(>|Chi|)
## 1 -10861
## 2 -10864  6.87  1    0.0088 **
## ---
## Signif. codes:  0 '***' 0.001 '**' 0.01 '*' 0.05 '.' 0.1 ' ' 1

Play_mod <- update(Breed_mod, .~. + Play)
anova(Breed_mod, Play_mod)

## Analysis of Deviance Table
##  Cox model: response is  Surv(LEC, Event)
##  Model 1: ~(1 | DogID) + Trial + BreedGroup
##  Model 2: ~(1 | DogID) + Trial + BreedGroup + Play
##   loglik Chisq Df P(>|Chi|)
## 1 -10861
## 2 -10865  7.97  1    0.0047 **
## ---
## Signif. codes:  0 '***' 0.001 '**' 0.01 '*' 0.05 '.' 0.1 ' ' 1

#Forth step
CI_mod <- update(Age_mod, .~. + CI)
anova(Age_mod, CI_mod) #*

## Analysis of Deviance Table
##  Cox model: response is  Surv(LEC, Event)
##  Model 1: ~(1 | DogID) + Trial + BreedGroup + Age
##  Model 2: ~(1 | DogID) + Trial + BreedGroup + Age + CI
##   loglik Chisq Df P(>|Chi|)
```

```
## 1 -10856
## 2 -10851  10.1  1    0.0015 **
## ---
## Signif. codes:  0 '***' 0.001 '**' 0.01 '*' 0.05 '.' 0.1 ' ' 1

Greet_mod <- update(Age_mod, .~. + Greet)
anova(Age_mod, Greet_mod)

## Analysis of Deviance Table
##  Cox model: response is  Surv(LEC, Event)
##  Model 1: ~(1 | DogID) + Trial + BreedGroup + Age
##  Model 2: ~(1 | DogID) + Trial + BreedGroup + Age + Greet
##   loglik Chisq Df P(>|Chi|)
## 1 -10856
## 2 -10858  3.76  1     0.053 .
## ---
## Signif. codes:  0 '***' 0.001 '**' 0.01 '*' 0.05 '.' 0.1 ' ' 1

Play_mod <- update(Age_mod, .~. + Play)
anova(Age_mod, Play_mod)

## Analysis of Deviance Table
##  Cox model: response is  Surv(LEC, Event)
##  Model 1: ~(1 | DogID) + Trial + BreedGroup + Age
##  Model 2: ~(1 | DogID) + Trial + BreedGroup + Age + Play
##   loglik Chisq Df P(>|Chi|)
## 1 -10856
## 2 -10857  3.65  1     0.056 .
## ---
## Signif. codes:  0 '***' 0.001 '**' 0.01 '*' 0.05 '.' 0.1 ' ' 1

#Fifth step
Greet_mod <- update(CI_mod, .~. + Greet)
anova(CI_mod, Greet_mod)

## Analysis of Deviance Table
##  Cox model: response is  Surv(LEC, Event)
##  Model 1: ~(1 | DogID) + Trial + BreedGroup + Age + CI
##  Model 2: ~(1 | DogID) + Trial + BreedGroup + Age + CI + Greet
##   loglik Chisq Df P(>|Chi|)
## 1 -10851
## 2 -10852  2.78  1     0.095 .
## ---
## Signif. codes:  0 '***' 0.001 '**' 0.01 '*' 0.05 '.' 0.1 ' ' 1

Play_mod <- update(CI_mod, .~. + Play)
anova(CI_mod, Play_mod)

## Analysis of Deviance Table
##  Cox model: response is  Surv(LEC, Event)
##  Model 1: ~(1 | DogID) + Trial + BreedGroup + Age + CI
##  Model 2: ~(1 | DogID) + Trial + BreedGroup + Age + CI + Play
##   loglik Chisq Df P(>|Chi|)
## 1 -10851
## 2 -10852  2.88  1      0.09 .
## ---
## Signif. codes:  0 '***' 0.001 '**' 0.01 '*' 0.05 '.' 0.1 ' ' 1

summary(Play_mod) #*
```

```
## Cox mixed-effects model fit by maximum likelihood
##   Data: EC_data
##   events, n = 1722, 1875
##   Iterations= 15 91
##                     NULL Integrated Fitted
## Log-likelihood -11640     -10852 -10594
##
##                      Chisq  df p  AIC  BIC
## Integrated loglik  1576   20 0 1536 1427
##  Penalized loglik  2093 130 0 1833 1124
##
## Model:  Surv(LEC, Event) ~ (1 | DogID) + Trial + BreedGroup + Age + CI +      Play
## Fixed coefficients
##                         coef exp(coef) se(coef)     z       p
## Trial2              0.05155    1.0529 0.135161  0.38 7.0e-01
## Trial3              0.18101    1.1984 0.133714  1.35 1.8e-01
## Trial4              0.42467    1.5291 0.133549  3.18 1.5e-03
## Trial5              0.62033    1.8595 0.135456  4.58 4.7e-06
## Trial6              0.64176    1.8998 0.134994  4.75 2.0e-06
## Trial7              0.48484    1.6239 0.135408  3.58 3.4e-04
## Trial8              0.65621    1.9275 0.135072  4.86 1.2e-06
## Trial9              0.69865    2.0110 0.137392  5.09 3.7e-07
## Trial10             0.63043    1.8784 0.136596  4.62 3.9e-06
## Trial11             0.70013    2.0140 0.137207  5.10 3.3e-07
## Trial12             0.77089    2.1617 0.136591  5.64 1.7e-08
## Trial13             0.84302    2.3234 0.139037  6.06 1.3e-09
## Trial14             0.57369    1.7748 0.137465  4.17 3.0e-05
## Trial15             0.72470    2.0641 0.138162  5.25 1.6e-07
## BreedGroupMixed    -0.01015    0.9899 0.251391 -0.04 9.7e-01
## BreedGroupNoncoop -1.08678    0.3373 0.298336 -3.64 2.7e-04
## Age                -0.01428    0.9858 0.002707 -5.27 1.3e-07
## CI                  0.05381    1.0553 0.018295  2.94 3.3e-03
## Play1               0.51487    1.6734 0.226305  2.28 2.3e-02
##
## Random effects
##  Group Variable  Std Dev Variance
##  DogID Intercept 1.051   1.106

#Sixth step
Greet_mod <- update(Play_mod, .~. + Greet)
anova(Play_mod, Greet_mod)

## Analysis of Deviance Table
##  Cox model: response is  Surv(LEC, Event)
##  Model 1: ~(1 | DogID) + Trial + BreedGroup + Age + CI + Play
##  Model 2: ~(1 | DogID) + Trial + BreedGroup + Age + CI + Play + Greet
##   loglik Chisq Df P(>|Chi|)
## 1 -10852
## 2 -10853  2.21  1      0.14

#FINAL model
EC_mod <- coxme(Surv(LEC, Event) ~ CI + BreedGroup + Age + Play + Trial + (1|DogID), data =
EC_data)
summary(EC_mod)

## Cox mixed-effects model fit by maximum likelihood
##   Data: EC_data
```

```
##   events, n = 1722, 1875
##   Iterations= 15 91
##                    NULL Integrated Fitted
## Log-likelihood -11640     -10852 -10594
##
##                     Chisq  df p  AIC  BIC
## Integrated loglik  1576  20 0 1536 1427
##  Penalized loglik  2093 130 0 1833 1124
##
## Model:  Surv(LEC, Event) ~ CI + BreedGroup + Age + Play + Trial + (1 |      DogID)
## Fixed coefficients
##                         coef exp(coef) se(coef)     z       p
## CI                   0.05381    1.0553 0.018295  2.94 3.3e-03
## BreedGroupMixed     -0.01015    0.9899 0.251391 -0.04 9.7e-01
## BreedGroupNoncoop -1.08678    0.3373 0.298336 -3.64 2.7e-04
## Age                 -0.01428    0.9858 0.002707 -5.27 1.3e-07
## Play1                0.51487    1.6734 0.226305  2.28 2.3e-02
## Trial2               0.05155    1.0529 0.135161  0.38 7.0e-01
## Trial3               0.18101    1.1984 0.133714  1.35 1.8e-01
## Trial4               0.42467    1.5291 0.133549  3.18 1.5e-03
## Trial5               0.62033    1.8595 0.135456  4.58 4.7e-06
## Trial6               0.64176    1.8998 0.134994  4.75 2.0e-06
## Trial7               0.48484    1.6239 0.135408  3.58 3.4e-04
## Trial8               0.65621    1.9275 0.135072  4.86 1.2e-06
## Trial9               0.69865    2.0110 0.137392  5.09 3.7e-07
## Trial10              0.63043    1.8784 0.136596  4.62 3.9e-06
## Trial11              0.70013    2.0140 0.137207  5.10 3.3e-07
## Trial12              0.77089    2.1617 0.136591  5.64 1.7e-08
## Trial13              0.84302    2.3234 0.139037  6.06 1.3e-09
## Trial14              0.57369    1.7748 0.137465  4.17 3.0e-05
## Trial15              0.72470    2.0641 0.138162  5.25 1.6e-07
##
## Random effects
##  Group Variable  Std Dev Variance
##  DogID Intercept 1.051   1.106
```

```
pairs(emmeans(EC_mod, ~BreedGroup))
```

```
##  contrast         estimate    SE  df z.ratio p.value
##  Coop - Mixed      0.0102 0.251 Inf 0.040   0.9991
##  Coop - Noncoop    1.0868 0.298 Inf 3.643   0.0008
##  Mixed - Noncoop   1.0766 0.273 Inf 3.949   0.0002
##
## Results are averaged over the levels of: Play, Trial
## Results are given on the log (not the response) scale.
## P value adjustment: tukey method for comparing a family of 3 estimates
```

```
pairs(emmeans(EC_mod, ~Trial))
```

```
##  contrast estimate    SE  df z.ratio p.value
##  1 - 2     -0.0515 0.135 Inf -0.381  1.0000
##  1 - 3     -0.1810 0.134 Inf -1.354  0.9909
##  1 - 4     -0.4247 0.134 Inf -3.180  0.0943
##  1 - 5     -0.6203 0.135 Inf -4.580  0.0005
##  1 - 6     -0.6418 0.135 Inf -4.754  0.0002
##  1 - 7     -0.4848 0.135 Inf -3.581  0.0268
##  1 - 8     -0.6562 0.135 Inf -4.858  0.0001
```

```
##  1 - 9     -0.6987 0.137 Inf -5.085  <.0001
##  1 - 10    -0.6304 0.137 Inf -4.615  0.0004
##  1 - 11    -0.7001 0.137 Inf -5.103  <.0001
##  1 - 12    -0.7709 0.137 Inf -5.644  <.0001
##  1 - 13    -0.8430 0.139 Inf -6.063  <.0001
##  1 - 14    -0.5737 0.137 Inf -4.173  0.0028
##  1 - 15    -0.7247 0.138 Inf -5.245  <.0001
##  2 - 3     -0.1295 0.135 Inf -0.960  0.9998
##  2 - 4     -0.3731 0.135 Inf -2.769  0.2636
##  2 - 5     -0.5688 0.135 Inf -4.207  0.0024
##  2 - 6     -0.5902 0.136 Inf -4.325  0.0014
##  2 - 7     -0.4333 0.135 Inf -3.202  0.0887
##  2 - 8     -0.6047 0.137 Inf -4.407  0.0010
##  2 - 9     -0.6471 0.138 Inf -4.682  0.0003
##  2 - 10    -0.5789 0.137 Inf -4.220  0.0023
##  2 - 11    -0.6486 0.138 Inf -4.689  0.0003
##  2 - 12    -0.7193 0.138 Inf -5.211  <.0001
##  2 - 13    -0.7915 0.139 Inf -5.700  <.0001
##  2 - 14    -0.5221 0.138 Inf -3.772  0.0135
##  2 - 15    -0.6732 0.139 Inf -4.839  0.0001
##  3 - 4     -0.2437 0.134 Inf -1.815  0.8927
##  3 - 5     -0.4393 0.135 Inf -3.249  0.0773
##  3 - 6     -0.4608 0.136 Inf -3.390  0.0502
##  3 - 7     -0.3038 0.135 Inf -2.244  0.6324
##  3 - 8     -0.4752 0.136 Inf -3.494  0.0358
##  3 - 9     -0.5176 0.137 Inf -3.769  0.0137
##  3 - 10    -0.4494 0.137 Inf -3.283  0.0698
##  3 - 11    -0.5191 0.137 Inf -3.783  0.0130
##  3 - 12    -0.5899 0.137 Inf -4.293  0.0017
##  3 - 13    -0.6620 0.139 Inf -4.764  0.0002
##  3 - 14    -0.3927 0.139 Inf -2.831  0.2301
##  3 - 15    -0.5437 0.138 Inf -3.937  0.0072
##  4 - 5     -0.1957 0.134 Inf -1.456  0.9820
##  4 - 6     -0.2171 0.134 Inf -1.615  0.9557
##  4 - 7     -0.0602 0.134 Inf -0.448  1.0000
##  4 - 8     -0.2315 0.135 Inf -1.710  0.9305
##  4 - 9     -0.2740 0.137 Inf -2.003  0.7968
##  4 - 10    -0.2058 0.135 Inf -1.523  0.9732
##  4 - 11    -0.2755 0.137 Inf -2.010  0.7928
##  4 - 12    -0.3462 0.136 Inf -2.545  0.4076
##  4 - 13    -0.4184 0.137 Inf -3.046  0.1359
##  4 - 14    -0.1490 0.137 Inf -1.089  0.9991
##  4 - 15    -0.3000 0.137 Inf -2.186  0.6751
##  5 - 6     -0.0214 0.136 Inf -0.158  1.0000
##  5 - 7      0.1355 0.134 Inf  1.007  0.9996
##  5 - 8     -0.0359 0.135 Inf -0.266  1.0000
##  5 - 9     -0.0783 0.136 Inf -0.574  1.0000
##  5 - 10    -0.0101 0.136 Inf -0.074  1.0000
##  5 - 11    -0.0798 0.137 Inf -0.584  1.0000
##  5 - 12    -0.1506 0.137 Inf -1.103  0.9989
##  5 - 13    -0.2227 0.138 Inf -1.611  0.9568
##  5 - 14     0.0466 0.137 Inf  0.340  1.0000
##  5 - 15    -0.1044 0.137 Inf -0.760  1.0000
##  6 - 7      0.1569 0.135 Inf  1.160  0.9981
##  6 - 8     -0.0144 0.136 Inf -0.106  1.0000
##  6 - 9     -0.0569 0.138 Inf -0.413  1.0000
##  6 - 10     0.0113 0.136 Inf  0.083  1.0000
```

```
##  6 - 11    -0.0584 0.138 Inf -0.423  1.0000
##  6 - 12    -0.1291 0.137 Inf -0.941  0.9998
##  6 - 13    -0.2013 0.138 Inf -1.456  0.9820
##  6 - 14     0.0681 0.138 Inf  0.493  1.0000
##  6 - 15    -0.0829 0.139 Inf -0.599  1.0000
##  7 - 8     -0.1714 0.136 Inf -1.262  0.9955
##  7 - 9     -0.2138 0.136 Inf -1.568  0.9654
##  7 - 10    -0.1456 0.135 Inf -1.078  0.9992
##  7 - 11    -0.2153 0.136 Inf -1.579  0.9634
##  7 - 12    -0.2860 0.136 Inf -2.101  0.7347
##  7 - 13    -0.3582 0.137 Inf -2.608  0.3642
##  7 - 14    -0.0888 0.138 Inf -0.644  1.0000
##  7 - 15    -0.2399 0.138 Inf -1.743  0.9197
##  8 - 9     -0.0424 0.137 Inf -0.310  1.0000
##  8 - 10     0.0258 0.137 Inf  0.189  1.0000
##  8 - 11    -0.0439 0.138 Inf -0.319  1.0000
##  8 - 12    -0.1147 0.136 Inf -0.841  1.0000
##  8 - 13    -0.1868 0.138 Inf -1.352  0.9911
##  8 - 14     0.0825 0.138 Inf  0.597  1.0000
##  8 - 15    -0.0685 0.138 Inf -0.496  1.0000
##  9 - 10     0.0682 0.137 Inf  0.499  1.0000
##  9 - 11    -0.0015 0.138 Inf -0.011  1.0000
##  9 - 12    -0.0722 0.137 Inf -0.526  1.0000
##  9 - 13    -0.1444 0.139 Inf -1.039  0.9994
##  9 - 14     0.1250 0.139 Inf  0.900  0.9999
##  9 - 15    -0.0260 0.139 Inf -0.187  1.0000
##  10 - 11   -0.0697 0.137 Inf -0.508  1.0000
##  10 - 12   -0.1405 0.136 Inf -1.030  0.9995
##  10 - 13   -0.2126 0.138 Inf -1.543  0.9700
##  10 - 14    0.0567 0.138 Inf  0.411  1.0000
##  10 - 15   -0.0943 0.138 Inf -0.684  1.0000
##  11 - 12   -0.0708 0.138 Inf -0.514  1.0000
##  11 - 13   -0.1429 0.140 Inf -1.023  0.9995
##  11 - 14    0.1264 0.139 Inf  0.908  0.9999
##  11 - 15   -0.0246 0.139 Inf -0.177  1.0000
##  12 - 13   -0.0721 0.138 Inf -0.521  1.0000
##  12 - 14    0.1972 0.138 Inf  1.432  0.9846
##  12 - 15    0.0462 0.138 Inf  0.334  1.0000
##  13 - 14    0.2693 0.139 Inf  1.931  0.8378
##  13 - 15    0.1183 0.140 Inf  0.847  1.0000
##  14 - 15   -0.1510 0.139 Inf -1.084  0.9991
##
## Results are averaged over the levels of: BreedGroup, Play
## Results are given on the log (not the response) scale.
## P value adjustment: tukey method for comparing a family of 15 estimates
```

```
#Figures
library(survival)
library(survminer)

CI <- survfit(Surv(LEC, Event) ~ CI_fig, data = EC_data)
ggsurvplot(CI, fun="event", conf.int = T, censor = F, xlim=c(0,15),break.x.by=5,
           linetype = c("strata"),
           xlab="Latency to form eye contact (s)",ylab="Ratio of dogs that formed \n eye co
ntact",
           legend.title = "Cephalic index", legend.labs = c("43.5-49.9", "50.0-55.3","55.4-
74.7"),
```

```
            font.x=c(12),font.y=c(12),font.tickslab=c(10),font.legend=c(10),
            ggtheme = theme_bw())
```

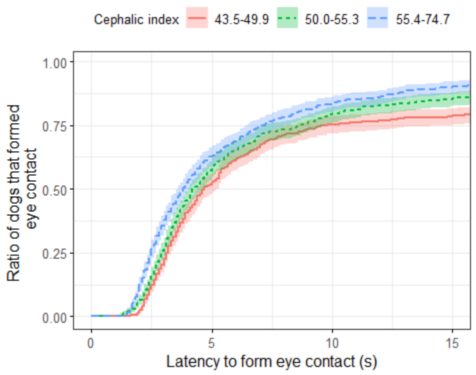


```
Breed <- survfit(Surv(LEC, Event) ~ BreedGroup, data = EC_data)
ggsurvplot(Breed, fun="event", conf.int = T, censor = F, xlim=c(0,15),break.x.by=5,
           linetype = c("strata"),
           xlab="Latency to form eye contact (s)",ylab="Ratio of dogs that formed \n eye co
ntact",
           legend.title = "Breed function", legend.labs = c("Cooperative", "Mixed","Non-coo
perative"),
           font.x=c(12),font.y=c(12),font.tickslab=c(10),font.legend=c(10),
           ggtheme = theme_bw())
```

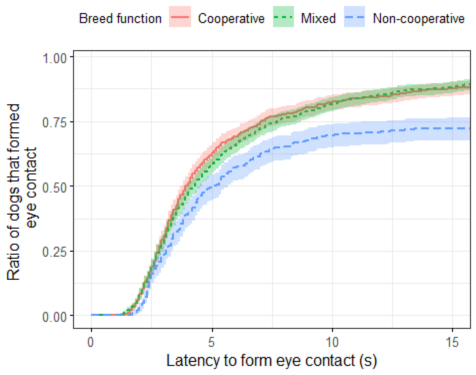


```
Age <- survfit(Surv(LEC, Event) ~ Age_fig, data = EC_data)
ggsurvplot(Age, fun="event", conf.int = T, censor = F, xlim=c(0,15),break.x.by=5,
           linetype = c("strata"),
           xlab="Latency to form eye contact (s)",ylab="Ratio of dogs that formed \n eye co
ntact",
           legend.title = "Age (month)", legend.labs = c('31.4-79.4','79.8-126.1','126.8-17
4.5'),
           font.x=c(12),font.y=c(12),font.tickslab=c(10),font.legend=c(10),
           ggtheme = theme_bw())
```

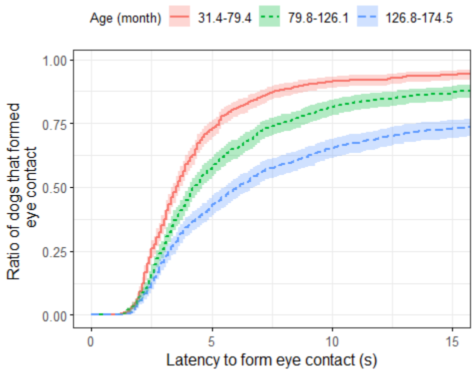


```
Play <- survfit(Surv(LEC, Event) ~ Play, data = EC_data)
ggsurvplot(Play, fun="event", conf.int = T, censor = F, xlim=c(0,15), break.x.by=5,
           linetype = c("strata"),
           xlab="Latency to form eye contact (s)",ylab="Ratio of dogs that formed \n eye co
ntact",
           legend.title = "Human-directed playfulness", legend.labs = c("Low", "High"),
           font.x=c(12),font.y=c(12),font.tickslab=c(10),font.legend=c(10),
           ggtheme = theme_bw())
```

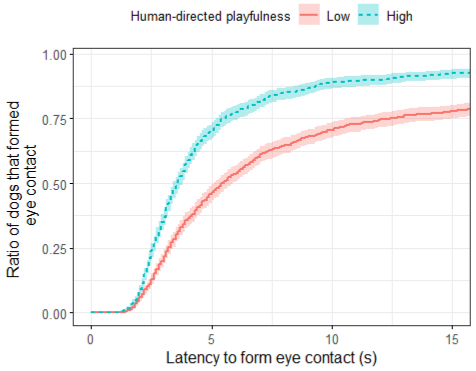


```
#Effect of experimenter
EC_mod_E <- update(EC_mod, .~. + Exp)
anova(EC_mod,EC_mod_E)

## Analysis of Deviance Table
##  Cox model: response is  Surv(LEC, Event)
##  Model 1: ~CI + BreedGroup + Age + Play + Trial + (1 | DogID)
##  Model 2: ~CI + BreedGroup + Age + Play + Trial + (1 | DogID) + Exp
##   loglik Chisq Df P(>|Chi|)
## 1 -10852
## 2 -10842  20.6  7    0.0044 **
## ---
## Signif. codes:  0 '***' 0.001 '**' 0.01 '*' 0.05 '.' 0.1 ' ' 1

pairs(emmeans(EC_mod_E, ~Exp))

##  contrast  estimate    SE  df z.ratio p.value
##  BR - BZs     0.259 0.560 Inf  0.463  0.9998
##  BR - DA      0.660 0.706 Inf  0.934  0.9827
##  BR - EA     -0.106 0.779 Inf -0.137  1.0000
##  BR - HV     -0.233 0.660 Inf -0.353  1.0000
##  BR - MS     -0.727 0.640 Inf -1.136  0.9489
##  BR - SB      0.101 0.576 Inf  0.175  1.0000
##  BR - SzD    -0.385 0.576 Inf -0.669  0.9978
##  BZs - DA     0.401 0.514 Inf  0.780  0.9942
```

```
##  BZs - EA    -0.366 0.626 Inf -0.585  0.9991
##  BZs - HV    -0.493 0.495 Inf -0.995  0.9752
##  BZs - MS    -0.986 0.488 Inf -2.022  0.4666
##  BZs - SB    -0.158 0.394 Inf -0.402  0.9999
##  BZs - SzD   -0.645 0.422 Inf -1.528  0.7925
##  DA - EA     -0.767 0.669 Inf -1.146  0.9466
##  DA - HV     -0.893 0.626 Inf -1.428  0.8447
##  DA - MS     -1.387 0.641 Inf -2.163  0.3741
##  DA - SB     -0.559 0.560 Inf -0.998  0.9749
##  DA - SzD    -1.046 0.594 Inf -1.759  0.6478
##  EA - HV     -0.127 0.690 Inf -0.184  1.0000
##  EA - MS     -0.620 0.717 Inf -0.865  0.9890
##  EA - SB      0.207 0.634 Inf  0.327  1.0000
##  EA - SzD    -0.279 0.683 Inf -0.408  0.9999
##  HV - MS     -0.494 0.604 Inf -0.818  0.9922
##  HV - SB      0.334 0.538 Inf  0.621  0.9986
##  HV - SzD    -0.152 0.544 Inf -0.280  1.0000
##  MS - SB      0.828 0.509 Inf  1.626  0.7344
##  MS - SzD     0.342 0.500 Inf  0.683  0.9974
##  SB - SzD    -0.486 0.444 Inf -1.096  0.9578
##
## Results are averaged over the levels of: BreedGroup, Play, Trial
## Results are given on the log (not the response) scale.
## P value adjustment: tukey method for comparing a family of 8 estimates

#Effect of sex
EC_mod_S <- update(EC_mod, .~. + Sex)
anova(EC_mod,EC_mod_S)

## Analysis of Deviance Table
##  Cox model: response is  Surv(LEC, Event)
##  Model 1: ~CI + BreedGroup + Age + Play + Trial + (1 | DogID)
##  Model 2: ~CI + BreedGroup + Age + Play + Trial + (1 | DogID) + Sex
##   loglik Chisq Df P(>|Chi|)
## 1 -10852
## 2 -10853  2.35  1      0.13

#Effect of neuter status
EC_mod_N <- update(EC_mod, .~. + Neutered)
anova(EC_mod,EC_mod_N)

## Analysis of Deviance Table
##  Cox model: response is  Surv(LEC, Event)
##  Model 1: ~CI + BreedGroup + Age + Play + Trial + (1 | DogID)
##  Model 2: ~CI + BreedGroup + Age + Play + Trial + (1 | DogID) + Neutered
##   loglik Chisq Df P(>|Chi|)
## 1 -10852
## 2 -10852  0.19  1      0.67

#Effect of weight
Weight_data <- subset(EC_data, !is.na(Weight)) # delete NAs
Weight_data$DogID <- factor(Weight_data$DogID)

EC_mod_W0 <- coxme(Surv(LEC, Event) ~ CI + BreedGroup + Age + Play + Trial + (1|DogID), dat
a = Weight_data) # final model without weight NAs
EC_mod_W <- update(EC_mod_W0, .~. + Weight)
anova(EC_mod_W0,EC_mod_W)
```

```
## Analysis of Deviance Table
##  Cox model: response is  Surv(LEC, Event)
##  Model 1: ~CI + BreedGroup + Age + Play + Trial + (1 | DogID)
##  Model 2: ~CI + BreedGroup + Age + Play + Trial + (1 | DogID) + Weight
##   loglik Chisq Df P(>|Chi|)
## 1 -10740
## 2 -10740  0.22  1      0.64
```

```
#Effect of previous dog school attendance
DogSchool_data <- subset(EC_data, !is.na(DogSchool)) # delete NAs
DogSchool_data$DogID <- factor(DogSchool_data$DogID)

EC_mod_DS0 <- coxme(Surv(LEC, Event) ~ CI + BreedGroup + Age + Play + Trial + (1|DogID), da
ta = DogSchool_data) # final model without dog school NAs
EC_mod_DS <- update(EC_mod_DS0, .~. + DogSchool)
anova(EC_mod_DS0,EC_mod_DS)
```

```
## Analysis of Deviance Table
##  Cox model: response is  Surv(LEC, Event)
##  Model 1: ~CI + BreedGroup + Age + Play + Trial + (1 | DogID)
##  Model 2: ~CI + BreedGroup + Age + Play + Trial + (1 | DogID) + DogSchool
##   loglik Chisq Df P(>|Chi|)
## 1  -9565
## 2  -9568  6.78  1    0.0092 **
## ---
## Signif. codes:  0 '***' 0.001 '**' 0.01 '*' 0.05 '.' 0.1 ' ' 1
```

```
#Interactions with trial
EC_mod_CI <- update(EC_mod, .~. + CI*Trial)
anova(EC_mod,EC_mod_CI)
```

```
## Analysis of Deviance Table
##  Cox model: response is  Surv(LEC, Event)
##  Model 1: ~CI + BreedGroup + Age + Play + Trial + (1 | DogID)
##  Model 2: ~CI + BreedGroup + Age + Play + Trial + (1 | DogID) + CI:Trial
##   loglik Chisq Df P(>|Chi|)
## 1 -10852
## 2 -10846    12 14      0.61
```

```
EC_mod_Breed <- update(EC_mod, .~. + BreedGroup*Trial)
anova(EC_mod,EC_mod_Breed)
```

```
## Analysis of Deviance Table
##  Cox model: response is  Surv(LEC, Event)
##  Model 1: ~CI + BreedGroup + Age + Play + Trial + (1 | DogID)
##  Model 2: ~CI + BreedGroup + Age + Play + Trial + (1 | DogID) + BreedGroup:Trial
##   loglik Chisq Df P(>|Chi|)
## 1 -10852
## 2 -10842  20.3 28      0.85
```

```
EC_mod_Age <- update(EC_mod, .~. + Age*Trial)
anova(EC_mod,EC_mod_Age)
```

```
## Analysis of Deviance Table
##  Cox model: response is  Surv(LEC, Event)
##  Model 1: ~CI + BreedGroup + Age + Play + Trial + (1 | DogID)
##  Model 2: ~CI + BreedGroup + Age + Play + Trial + (1 | DogID) + Age:Trial
##   loglik Chisq Df P(>|Chi|)
```

```
## 1 -10852
## 2 -10843  18.8 14      0.17
```

```
EC_mod_Play <- update(EC_mod, .~. + Play*Trial)
anova(EC_mod,EC_mod_Play)
```

```
## Analysis of Deviance Table
##  Cox model: response is  Surv(LEC, Event)
##  Model 1: ~CI + BreedGroup + Age + Play + Trial + (1 | DogID)
##  Model 2: ~CI + BreedGroup + Age + Play + Trial + (1 | DogID) + Play:Trial
##   loglik Chisq Df P(>|Chi|)
## 1 -10852
## 2 -10849  6.85 14      0.94
```

b. Sociability tests

i. Greeting

```
library(MuMIn)
```

```
## Registered S3 methods overwritten by 'MuMIn':
##   method         from
##   formula.coxme coxme
##   logLik.coxme  coxme
##   logLik.lmekin coxme
```

```
options(na.action = na.fail)

Greet_model <- glm(Greet ~ CI + BreedGroup + Age, family=binomial(link="logit"), data=Soc_d
ata)
dredge(Greet_model)
```

```
## Fixed term is "(Intercept)"
```

```
## Global model call: glm(formula = Greet ~ CI + BreedGroup + Age, family = binomial(link =
"logit"),
##     data = Soc_data)
## ---
## Model selection table
##    (Intrc)       Age BrdGr      CI df logLik  AICc delta weight
## 2  1.31300 -0.009536                2 -82.86 169.8  0.00  0.263
## 4  2.03200 -0.011100     +          4 -80.79 169.9  0.08  0.252
## 6 -0.06499 -0.010130       0.02690  3 -82.51 171.2  1.39  0.131
## 8  0.52800 -0.011730     + 0.02945  5 -80.37 171.2  1.41  0.130
## 1  0.33920                          1 -84.87 171.8  1.95  0.099
## 3  0.80230               +          3 -83.31 172.8  3.00  0.059
## 5 -0.57630                 0.01712  2 -84.72 173.5  3.71  0.041
## 7 -0.19990               + 0.01885  4 -83.13 174.6  4.77  0.024
## Models ranked by AICc(x)
```

```
Greet_FIN_model <- glm(Greet ~ Age, family=binomial(link="logit"), data=Soc_data)
summary(Greet_FIN_model)
```

```
##
## Call:
## glm(formula = Greet ~ Age, family = binomial(link = "logit"),
##     data = Soc_data)
##
```

```
## Deviance Residuals:
##    Min      1Q  Median      3Q     Max
## -1.605  -1.220   0.816   1.074   1.284
##
## Coefficients:
##             Estimate Std. Error z value Pr(>|z|)
## (Intercept)  1.31340    0.53521    2.45    0.014 *
## Age         -0.00954    0.00485   -1.97    0.049 *
## ---
## Signif. codes:  0 '***' 0.001 '**' 0.01 '*' 0.05 '.' 0.1 ' ' 1
##
## (Dispersion parameter for binomial family taken to be 1)
##
##     Null deviance: 169.74  on 124  degrees of freedom
## Residual deviance: 165.73  on 123  degrees of freedom
## AIC: 169.7
##
## Number of Fisher Scoring iterations: 4
```

```
ggplot(Soc_data_fig, aes(Age, Greet))+
  ylim(0,1)+
  geom_smooth(method = "glm", method.args = list(family = "binomial"),
              color="black",size=0.6, fill="turquoise3")+
  xlab("Age (month)") + ylab("Probability of \n greeting the experimenter")+
  theme_bw()+
  theme(axis.text.x = element_text(size = 10), axis.title.x = element_text(size = 12),
        axis.text.y = element_text(size = 10), axis.title.y = element_text(size = 12))
```

```
## `geom_smooth()` using formula 'y ~ x'
```

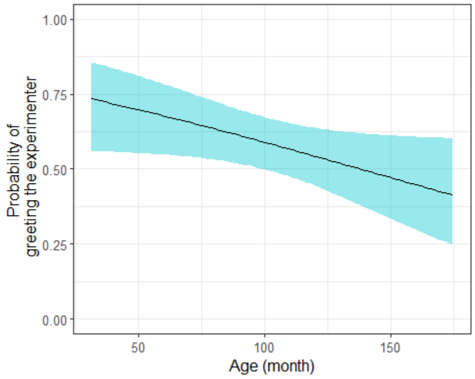

ii. Playfulness

```
Play_model <- glm(Play ~ CI + BreedGroup + Age, family=binomial(link="logit"), data=Soc_dat
a)
dredge(Play_model)
```

```
## Fixed term is "(Intercept)"
```

```
## Global model call: glm(formula = Play ~ CI + BreedGroup + Age, family = binomial(link =
"logit"),
##     data = Soc_data)
## ---
## Model selection table
##    (Intrc)      Age BrdGr       CI df logLik  AICc delta weight
## 2  1.86700 -0.01932                2 -78.69 161.5  0.00  0.488
## 4  2.50800 -0.02104     +          4 -77.23 162.8  1.31  0.254
```

```
## 6  1.76800 -0.01937        0.001930  3 -78.69 163.6  2.10  0.171
## 8  2.27400 -0.02116     +  0.004612  5 -77.22 164.9  3.46  0.087
## 1 -0.08004                           1 -86.54 175.1 13.64  0.001
## 5  0.73900               -0.015300  2 -86.42 176.9 15.45  0.000
## 3  0.19110              +            3 -85.86 177.9 16.44  0.000
## 7  0.97380              + -0.014690  4 -85.75 179.8 18.34  0.000
## Models ranked by AICc(x)
```

```
Play_FIN_model <- glm(Play ~ Age, family=binomial(link="logit"), data=Soc_data)
summary(Play_FIN_model)
```

```
##
## Call:
## glm(formula = Play ~ Age, family = binomial(link = "logit"),
##     data = Soc_data)
##
## Deviance Residuals:
##    Min      1Q  Median      3Q     Max
## -1.700  -0.968  -0.697   1.042   1.753
##
## Coefficients:
##               Estimate Std. Error z value Pr(>|z|)
## (Intercept)  1.86710    0.55794    3.35  0.00082 ***
## Age         -0.01932    0.00518   -3.73  0.00019 ***
## ---
## Signif. codes:  0 '***' 0.001 '**' 0.01 '*' 0.05 '.' 0.1 ' ' 1
##
## (Dispersion parameter for binomial family taken to be 1)
##
##     Null deviance: 173.09  on 124  degrees of freedom
## Residual deviance: 157.39  on 123  degrees of freedom
## AIC: 161.4
##
## Number of Fisher Scoring iterations: 4
```

```
ggplot(Soc_data_fig, aes(Age, Play))+
  ylim(0,1) +
  geom_smooth(method = "glm", method.args = list(family = "binomial"),
              color="black",size=0.6, fill="turquoise3")+
  xlab("Age (month)") + ylab("Probability of \n playing with the experimenter")+
  theme_bw()+
  theme(axis.text.x = element_text(size = 10), axis.title.x = element_text(size = 12),
        axis.text.y = element_text(size = 10), axis.title.y = element_text(size = 12))
```

```
## `geom_smooth()` using formula 'y ~ x'
```

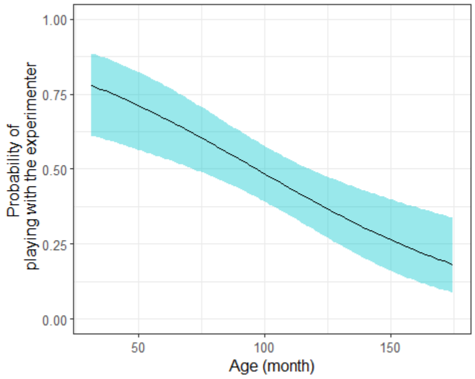

3. Trial analysis

a. 1. eye contact

```
EClec_data <- subset(EC_data, Trial==1)

EC_mod <- coxme(Surv(LEC, Event) ~ Age + Play + (1|DogID), data = EClec_data)
summary(EC_mod)

## Cox mixed-effects model fit by maximum likelihood
##   Data: EClec_data
##   events, n = 123, 125
##   Iterations= 2 15
##                     NULL Integrated Fitted
## Log-likelihood -481.2     -471.9 -455.8
##
##                       Chisq    df          p   AIC    BIC
## Integrated loglik 18.48  3.00 3.496e-04 12.48   4.05
##  Penalized loglik 50.84 16.92 2.973e-05 17.00 -30.58
##
## Model:  Surv(LEC, Event) ~ Age + Play + (1 | DogID)
## Fixed coefficients
##             coef exp(coef) se(coef)     z      p
## Age   -0.007437    0.9926 0.002872 -2.59 0.0096
## Play1  0.524260    1.6892 0.223974  2.34 0.0190
##
## Random effects
##  Group Variable  Std Dev Variance
##  DogID Intercept 0.3997  0.1598
```

b. 1-2. eye contact

```
EClec_data <- subset(EC_data, Trial==1 | Trial==2)
EClec_data$Trial <- factor(EClec_data$Trial,levels = c('1','2'))

EC_mod <- coxme(Surv(LEC, Event) ~ BreedGroup + Age + Play + (1|DogID), data = EClec_data)
summary(EC_mod)

## Cox mixed-effects model fit by maximum likelihood
##   Data: EClec_data
##   events, n = 243, 250
##   Iterations= 7 46
##                    NULL Integrated Fitted
## Log-likelihood -1126      -1079 -972.4
##
##                       Chisq    df p    AIC     BIC
## Integrated loglik  93.18  5.00 0  83.18   65.71
##  Penalized loglik 306.30 80.76 0 144.79 -137.30
##
## Model:  Surv(LEC, Event) ~ BreedGroup + Age + Play + (1 | DogID)
## Fixed coefficients
##                         coef exp(coef) se(coef)     z       p
## BreedGroupMixed   -0.15807    0.8538 0.266786 -0.59 5.5e-01
## BreedGroupNoncoop -1.08842    0.3367 0.327420 -3.32 8.9e-04
## Age               -0.01542    0.9847 0.003245 -4.75 2.0e-06
## Play1              0.56497    1.7594 0.249283  2.27 2.3e-02
```

```
##
## Random effects
##  Group Variable  Std Dev Variance
##  DogID Intercept 1.012   1.024

pairs(emmeans(EC_mod, ~BreedGroup))

##  contrast         estimate    SE  df z.ratio p.value
##  Coop - Mixed        0.158 0.267 Inf 0.592   0.8242
##  Coop - Noncoop      1.088 0.327 Inf 3.324   0.0026
##  Mixed - Noncoop     0.930 0.303 Inf 3.069   0.0061
##
## Results are averaged over the levels of: Play
## Results are given on the log (not the response) scale.
## P value adjustment: tukey method for comparing a family of 3 estimates
```

c. 1-3. eye contact

```
EClec_data <- subset(EC_data, Trial==1 | Trial==2 | Trial==3)
EClec_data$Trial <- factor(EClec_data$Trial,levels = c('1','2','3'))

EC_mod <- coxme(Surv(LEC, Event) ~ BreedGroup + Age + (1|DogID), data = EClec_data)
summary(EC_mod)

## Cox mixed-effects model fit by maximum likelihood
##   Data: EClec_data
##   events, n = 361, 375
##   Iterations= 18 106
##                   NULL Integrated Fitted
## Log-likelihood -1826      -1725  -1561
##
##                    Chisq    df p   AIC    BIC
## Integrated loglik 202.6   4.0 0 194.6 179.03
##  Penalized loglik 530.5 103.9 0 322.7 -81.19
##
## Model:  Surv(LEC, Event) ~ BreedGroup + Age + (1 | DogID)
## Fixed coefficients
##                       coef exp(coef) se(coef)     z       p
## BreedGroupMixed   -0.27227    0.7617 0.311295 -0.87 3.8e-01
## BreedGroupNoncoop -1.50989    0.2209 0.377706 -4.00 6.4e-05
## Age               -0.02038    0.9798 0.003341 -6.10 1.1e-09
##
## Random effects
##  Group Variable  Std Dev Variance
##  DogID Intercept 1.332   1.776

pairs(emmeans(EC_mod, ~BreedGroup))

##  contrast         estimate    SE  df z.ratio p.value
##  Coop - Mixed        0.272 0.311 Inf 0.875   0.6563
##  Coop - Noncoop      1.510 0.378 Inf 3.998   0.0002
##  Mixed - Noncoop     1.238 0.357 Inf 3.471   0.0015
##
## Results are given on the log (not the response) scale.
## P value adjustment: tukey method for comparing a family of 3 estimates
```

d. 1-4. eye contact

```
EClec_data <- subset(EC_data, Trial==1 | Trial==2 | Trial==3 | Trial==4)
EClec_data$Trial <- factor(EClec_data$Trial,levels = c('1','2','3','4'))

EC_mod <- coxme(Surv(LEC, Event) ~ Trial + CI + BreedGroup + Age + Play + (1|DogID), data =
EClec_data)
summary(EC_mod)

## Cox mixed-effects model fit by maximum likelihood
##   Data: EClec_data
##   events, n = 479, 500
##   Iterations= 14 87
##                     NULL Integrated Fitted
## Log-likelihood -2566      -2411   -2231
##
##                       Chisq    df p   AIC    BIC
## Integrated loglik 310.1   9.0 0 292.1 254.52
##  Penalized loglik 669.7 111.2 0 447.2 -16.92
##
## Model:  Surv(LEC, Event) ~ Trial + CI + BreedGroup + Age + Play + (1 |      DogID)
## Fixed coefficients
##                          coef exp(coef) se(coef)     z       p
## Trial2              -0.06040    0.9414 0.144802 -0.42 6.8e-01
## Trial3               0.19235    1.2121 0.138904  1.38 1.7e-01
## Trial4               0.33062    1.3918 0.140583  2.35 1.9e-02
## CI                   0.04840    1.0496 0.022457  2.16 3.1e-02
## BreedGroupMixed     -0.09542    0.9090 0.310150 -0.31 7.6e-01
## BreedGroupNoncoop -1.33061    0.2643 0.374501 -3.55 3.8e-04
## Age                 -0.01667    0.9835 0.003631 -4.59 4.4e-06
## Play1                0.58157    1.7889 0.282434  2.06 3.9e-02
##
## Random effects
##  Group Variable  Std Dev Variance
##  DogID Intercept 1.319   1.741

pairs(emmeans(EC_mod, ~Trial))

##  contrast estimate    SE  df z.ratio p.value
##  1 - 2      0.0604 0.145 Inf  0.417  0.9756
##  1 - 3     -0.1923 0.139 Inf -1.385  0.5089
##  1 - 4     -0.3306 0.141 Inf -2.352  0.0866
##  2 - 3     -0.2528 0.146 Inf -1.737  0.3045
##  2 - 4     -0.3910 0.147 Inf -2.669  0.0382
##  3 - 4     -0.1383 0.144 Inf -0.962  0.7708
##
## Results are averaged over the levels of: BreedGroup, Play
## Results are given on the log (not the response) scale.
## P value adjustment: tukey method for comparing a family of 4 estimates

pairs(emmeans(EC_mod, ~BreedGroup))

##  contrast        estimate    SE  df z.ratio p.value
##  Coop - Mixed      0.0954 0.310 Inf 0.308   0.9492
##  Coop - Noncoop    1.3306 0.374 Inf 3.553   0.0011
##  Mixed - Noncoop   1.2352 0.348 Inf 3.552   0.0011
##
```

```
## Results are averaged over the levels of: Trial, Play
## Results are given on the log (not the response) scale.
## P value adjustment: tukey method for comparing a family of 3 estimates
```

e. 1-5. eye contact

```
EClec_data <- subset(EC_data, Trial==1 | Trial==2 | Trial==3 | Trial==4 | Trial==5)
EClec_data$Trial <- factor(EClec_data$Trial,levels = c('1','2','3','4','5'))

EC_mod <- coxme(Surv(LEC, Event) ~ Trial + CI + BreedGroup + Age + (1|DogID), data = EClec_
data)
summary(EC_mod)

## Cox mixed-effects model fit by maximum likelihood
##   Data: EClec_data
##   events, n = 596, 625
##   Iterations= 19 111
##                   NULL Integrated Fitted
## Log-likelihood -3331      -3126  -2932
##
##                    Chisq    df p   AIC    BIC
## Integrated loglik 411.2   9.0 0 393.2 353.67
##  Penalized loglik 798.4 115.2 0 567.9  62.04
##
## Model:  Surv(LEC, Event) ~ Trial + CI + BreedGroup + Age + (1 | DogID)
## Fixed coefficients
##                        coef exp(coef) se(coef)     z       p
## Trial2             -0.01269    0.9874 0.142626 -0.09 9.3e-01
## Trial3              0.22101    1.2473 0.138264  1.60 1.1e-01
## Trial4              0.36279    1.4373 0.139702  2.60 9.4e-03
## Trial5              0.50808    1.6621 0.141928  3.58 3.4e-04
## CI                  0.04910    1.0503 0.022342  2.20 2.8e-02
## BreedGroupMixed    -0.16624    0.8468 0.310764 -0.53 5.9e-01
## BreedGroupNoncoop -1.36759    0.2547 0.368909 -3.71 2.1e-04
## Age                -0.01949    0.9807 0.003137 -6.21 5.1e-10
##
## Random effects
##  Group Variable  Std Dev Variance
##  DogID Intercept 1.331   1.772

pairs(emmeans(EC_mod, ~Trial))

##  contrast estimate    SE  df z.ratio p.value
##  1 - 2      0.0127 0.143 Inf  0.089  1.0000
##  1 - 3     -0.2210 0.138 Inf -1.598  0.4983
##  1 - 4     -0.3628 0.140 Inf -2.597  0.0709
##  1 - 5     -0.5081 0.142 Inf -3.580  0.0032
##  2 - 3     -0.2337 0.143 Inf -1.633  0.4761
##  2 - 4     -0.3755 0.144 Inf -2.609  0.0686
##  2 - 5     -0.5208 0.144 Inf -3.623  0.0027
##  3 - 4     -0.1418 0.142 Inf -0.996  0.8575
##  3 - 5     -0.2871 0.142 Inf -2.028  0.2525
##  4 - 5     -0.1453 0.142 Inf -1.023  0.8450
##
## Results are averaged over the levels of: BreedGroup
## Results are given on the log (not the response) scale.
## P value adjustment: tukey method for comparing a family of 5 estimates
```

```
pairs(emmeans(EC_mod, ~BreedGroup))

##  contrast        estimate    SE  df z.ratio p.value
##  Coop - Mixed       0.166 0.311 Inf 0.535   0.8541
##  Coop - Noncoop     1.368 0.369 Inf 3.707   0.0006
##  Mixed - Noncoop    1.201 0.351 Inf 3.420   0.0018
##
## Results are averaged over the levels of: Trial
## Results are given on the log (not the response) scale.
## P value adjustment: tukey method for comparing a family of 3 estimates
```

f. 1-6. eye contact

```
EClec_data <- subset(EC_data, Trial==1 | Trial==2 | Trial==3 | Trial==4 | Trial==5 | Trial=
=6)
EClec_data$Trial <- factor(EClec_data$Trial,levels = c('1','2','3','4','5','6'))

EC_mod <- coxme(Surv(LEC, Event) ~ Trial + CI + BreedGroup + Age + (1|DogID), data = EClec_
data)
summary(EC_mod)

## Cox mixed-effects model fit by maximum likelihood
##   Data: EClec_data
##   events, n = 713, 750
##   Iterations= 14 83
##                   NULL Integrated Fitted
## Log-likelihood -4120      -3852  -3643
##
##                   Chisq    df p   AIC   BIC
## Integrated loglik 535.6  10.0 0 515.6 469.9
##  Penalized loglik 953.2 118.8 0 715.5 172.4
##
## Model:  Surv(LEC, Event) ~ Trial + CI + BreedGroup + Age + (1 | DogID)
## Fixed coefficients
##                        coef exp(coef) se(coef)     z       p
## Trial2            -0.008425    0.9916 0.142333 -0.06 9.5e-01
## Trial3             0.208226    1.2315 0.138123  1.51 1.3e-01
## Trial4             0.410036    1.5069 0.138786  2.95 3.1e-03
## Trial5             0.532487    1.7032 0.140977  3.78 1.6e-04
## Trial6             0.662407    1.9395 0.139999  4.73 2.2e-06
## CI                 0.054399    1.0559 0.023252  2.34 1.9e-02
## BreedGroupMixed   -0.195173    0.8227 0.325805 -0.60 5.5e-01
## BreedGroupNoncoop -1.330911    0.2642 0.381251 -3.49 4.8e-04
## Age               -0.018997    0.9812 0.003128 -6.07 1.3e-09
##
## Random effects
##  Group Variable  Std Dev Variance
##  DogID Intercept 1.383   1.913

pairs(emmeans(EC_mod, ~Trial))

##  contrast estimate    SE  df z.ratio p.value
##  1 - 2      0.0084 0.142 Inf  0.059  1.0000
##  1 - 3     -0.2082 0.138 Inf -1.508  0.6594
##  1 - 4     -0.4100 0.139 Inf -2.954  0.0370
##  1 - 5     -0.5325 0.141 Inf -3.777  0.0022
##  1 - 6     -0.6624 0.140 Inf -4.732  <.0001
```

```
##  2 - 3     -0.2167 0.142 Inf -1.524  0.6487
##  2 - 4     -0.4185 0.143 Inf -2.935  0.0391
##  2 - 5     -0.5409 0.142 Inf -3.796  0.0020
##  2 - 6     -0.6708 0.144 Inf -4.666  <.0001
##  3 - 4     -0.2018 0.141 Inf -1.429  0.7096
##  3 - 5     -0.3243 0.141 Inf -2.305  0.1918
##  3 - 6     -0.4542 0.142 Inf -3.204  0.0170
##  4 - 5     -0.1225 0.140 Inf -0.872  0.9533
##  4 - 6     -0.2524 0.139 Inf -1.816  0.4550
##  5 - 6     -0.1299 0.140 Inf -0.925  0.9401
##
## Results are averaged over the levels of: BreedGroup
## Results are given on the log (not the response) scale.
## P value adjustment: tukey method for comparing a family of 6 estimates
```

```
pairs(emmeans(EC_mod, ~BreedGroup))
```

```
##  contrast        estimate    SE  df z.ratio p.value
##  Coop - Mixed       0.195 0.326 Inf 0.599   0.8206
##  Coop - Noncoop     1.331 0.381 Inf 3.491   0.0014
##  Mixed - Noncoop    1.136 0.365 Inf 3.110   0.0053
##
## Results are averaged over the levels of: Trial
## Results are given on the log (not the response) scale.
## P value adjustment: tukey method for comparing a family of 3 estimates
```

g. 1-7. eye contact

```
EClec_data <- subset(EC_data, Trial==1 | Trial==2 | Trial==3 | Trial==4 | Trial==5 | Trial=
=6 | Trial==7)
EClec_data$Trial <- factor(EClec_data$Trial,levels = c('1','2','3','4','5','6','7'))

EC_mod <- coxme(Surv(LEC, Event) ~ Trial + CI + BreedGroup + Age + (1|DogID), data = EClec_
data)
summary(EC_mod)
```

```
## Cox mixed-effects model fit by maximum likelihood
##   Data: EClec_data
##   events, n = 830, 875
##   Iterations= 9 58
##                   NULL Integrated Fitted
## Log-likelihood -4928      -4602  -4383
##
##                    Chisq    df p    AIC   BIC
## Integrated loglik   651  11.0 0 629.0 577.1
##  Penalized loglik  1088 121.2 0 846.1 273.9
##
## Model:  Surv(LEC, Event) ~ Trial + CI + BreedGroup + Age + (1 | DogID)
## Fixed coefficients
##                        coef exp(coef) se(coef)     z       p
## Trial2              0.02343    1.0237  0.14116  0.17 8.7e-01
## Trial3              0.22475    1.2520  0.13782  1.63 1.0e-01
## Trial4              0.44351    1.5582  0.13763  3.22 1.3e-03
## Trial5              0.58073    1.7873  0.14047  4.13 3.6e-05
## Trial6              0.71580    2.0458  0.13966  5.13 3.0e-07
## Trial7              0.44704    1.5637  0.14108  3.17 1.5e-03
## CI                  0.05208    1.0535  0.02351  2.22 2.7e-02
```

```
## BreedGroupMixed   -0.20801    0.8122  0.32723 -0.64 5.2e-01
## BreedGroupNoncoop -1.32032    0.2670  0.38502 -3.43 6.1e-04
## Age               -0.01874    0.9814  0.00309 -6.07 1.3e-09
##
## Random effects
##  Group Variable  Std Dev Variance
##  DogID Intercept 1.390   1.931
```

```
pairs(emmeans(EC_mod, ~Trial))
```

```
##  contrast estimate    SE  df z.ratio p.value
##  1 - 2     -0.0234 0.141 Inf -0.166  1.0000
##  1 - 3     -0.2248 0.138 Inf -1.631  0.6623
##  1 - 4     -0.4435 0.138 Inf -3.222  0.0217
##  1 - 5     -0.5807 0.140 Inf -4.134  0.0007
##  1 - 6     -0.7158 0.140 Inf -5.125  <.0001
##  1 - 7     -0.4470 0.141 Inf -3.169  0.0257
##  2 - 3     -0.2013 0.141 Inf -1.428  0.7867
##  2 - 4     -0.4201 0.140 Inf -2.992  0.0441
##  2 - 5     -0.5573 0.141 Inf -3.958  0.0015
##  2 - 6     -0.6924 0.142 Inf -4.873  <.0001
##  2 - 7     -0.4236 0.141 Inf -3.001  0.0429
##  3 - 4     -0.2188 0.140 Inf -1.568  0.7031
##  3 - 5     -0.3560 0.140 Inf -2.541  0.1447
##  3 - 6     -0.4911 0.141 Inf -3.491  0.0087
##  3 - 7     -0.2223 0.141 Inf -1.578  0.6968
##  4 - 5     -0.1372 0.139 Inf -0.988  0.9568
##  4 - 6     -0.2723 0.137 Inf -1.981  0.4267
##  4 - 7     -0.0035 0.139 Inf -0.025  1.0000
##  5 - 6     -0.1351 0.140 Inf -0.968  0.9608
##  5 - 7      0.1337 0.139 Inf  0.963  0.9619
##  6 - 7      0.2688 0.139 Inf  1.936  0.4565
##
## Results are averaged over the levels of: BreedGroup
## Results are given on the log (not the response) scale.
## P value adjustment: tukey method for comparing a family of 7 estimates
```

```
pairs(emmeans(EC_mod, ~BreedGroup))
```

```
##  contrast        estimate    SE  df z.ratio p.value
##  Coop - Mixed       0.208 0.327 Inf 0.636   0.8004
##  Coop - Noncoop     1.320 0.385 Inf 3.429   0.0018
##  Mixed - Noncoop    1.112 0.368 Inf 3.026   0.0070
##
## Results are averaged over the levels of: Trial
## Results are given on the log (not the response) scale.
## P value adjustment: tukey method for comparing a family of 3 estimates
```

h. 1-8. eye contact

```
EClec_data <- subset(EC_data, Trial==1 | Trial==2 | Trial==3 | Trial==4 | Trial==5 | Trial=
=6 | Trial==7 | Trial==8)
EClec_data$Trial <- factor(EClec_data$Trial,levels = c('1','2','3','4','5','6','7','8'))

EC_mod <- coxme(Surv(LEC, Event) ~ Trial + CI + BreedGroup + Age + (1|DogID), data = EClec_
data)
summary(EC_mod)
```

```
## Cox mixed-effects model fit by maximum likelihood
##   Data: EClec_data
##   events, n = 945, 1000
##   Iterations= 22 131
##                  NULL Integrated Fitted
## Log-likelihood -5744      -5368  -5145
##
##                      Chisq    df p   AIC   BIC
## Integrated loglik  752.5  12.0 0 728.5 670.3
##  Penalized loglik 1197.4 122.5 0 952.3 357.9
##
## Model:  Surv(LEC, Event) ~ Trial + CI + BreedGroup + Age + (1 | DogID)
## Fixed coefficients
##                        coef exp(coef) se(coef)     z       p
## Trial2             -0.00539    0.9946 0.139147 -0.04 9.7e-01
## Trial3              0.18412    1.2022 0.136168  1.35 1.8e-01
## Trial4              0.40880    1.5050 0.136405  3.00 2.7e-03
## Trial5              0.57654    1.7799 0.138505  4.16 3.1e-05
## Trial6              0.67547    1.9649 0.137953  4.90 9.8e-07
## Trial7              0.40214    1.4950 0.139371  2.89 3.9e-03
## Trial8              0.57074    1.7696 0.138566  4.12 3.8e-05
## CI                  0.05083    1.0521 0.022757  2.23 2.6e-02
## BreedGroupMixed    -0.19092    0.8262 0.314304 -0.61 5.4e-01
## BreedGroupNoncoop -1.28610    0.2763 0.371892 -3.46 5.4e-04
## Age                -0.01846    0.9817 0.002896 -6.38 1.8e-10
##
## Random effects
##  Group Variable  Std Dev Variance
##  DogID Intercept 1.334   1.781
```

```
pairs(emmeans(EC_mod, ~Trial))
```

```
##  contrast estimate    SE  df z.ratio p.value
##  1 - 2      0.0054 0.139 Inf   0.039  1.0000
##  1 - 3     -0.1841 0.136 Inf  -1.352  0.8786
##  1 - 4     -0.4088 0.136 Inf  -2.997  0.0552
##  1 - 5     -0.5765 0.139 Inf  -4.163  0.0008
##  1 - 6     -0.6755 0.138 Inf  -4.896  <.0001
##  1 - 7     -0.4021 0.139 Inf  -2.885  0.0755
##  1 - 8     -0.5707 0.139 Inf  -4.119  0.0010
##  2 - 3     -0.1895 0.139 Inf  -1.361  0.8750
##  2 - 4     -0.4142 0.139 Inf  -2.971  0.0595
##  2 - 5     -0.5819 0.140 Inf  -4.167  0.0008
##  2 - 6     -0.6809 0.141 Inf  -4.827  <.0001
##  2 - 7     -0.4075 0.140 Inf  -2.909  0.0708
##  2 - 8     -0.5761 0.142 Inf  -4.045  0.0014
##  3 - 4     -0.2247 0.139 Inf  -1.621  0.7378
##  3 - 5     -0.3924 0.139 Inf  -2.831  0.0875
##  3 - 6     -0.4913 0.140 Inf  -3.522  0.0102
##  3 - 7     -0.2180 0.140 Inf  -1.560  0.7745
##  3 - 8     -0.3866 0.141 Inf  -2.746  0.1092
##  4 - 5     -0.1677 0.138 Inf  -1.214  0.9281
##  4 - 6     -0.2667 0.137 Inf  -1.949  0.5166
##  4 - 7      0.0067 0.138 Inf   0.048  1.0000
##  4 - 8     -0.1619 0.140 Inf  -1.158  0.9437
##  5 - 6     -0.0989 0.139 Inf  -0.713  0.9966
##  5 - 7      0.1744 0.138 Inf   1.262  0.9128
```

```
##  5 - 8      0.0058 0.138 Inf  0.042  1.0000
##  6 - 7      0.2733 0.138 Inf  1.975  0.4988
##  6 - 8      0.1047 0.140 Inf  0.749  0.9955
##  7 - 8     -0.1686 0.141 Inf -1.195  0.9337
##
## Results are averaged over the levels of: BreedGroup
## Results are given on the log (not the response) scale.
## P value adjustment: tukey method for comparing a family of 8 estimates
```

```
pairs(emmeans(EC_mod, ~BreedGroup))
```

```
##  contrast        estimate    SE  df z.ratio p.value
##  Coop - Mixed       0.191 0.314 Inf 0.607   0.8161
##  Coop - Noncoop     1.286 0.372 Inf 3.458   0.0016
##  Mixed - Noncoop    1.095 0.351 Inf 3.123   0.0051
##
## Results are averaged over the levels of: Trial
## Results are given on the log (not the response) scale.
## P value adjustment: tukey method for comparing a family of 3 estimates
```

i. 1-9. eye contact

```
EClec_data <- subset(EC_data, Trial==1 | Trial==2 | Trial==3 | Trial==4 | Trial==5 | Trial=
=6 | Trial==7 | Trial==8 | Trial==9)
EClec_data$Trial <- factor(EClec_data$Trial,levels = c('1','2','3','4','5','6','7','8','9')
)

EC_mod <- coxme(Surv(LEC, Event) ~ Trial + CI + BreedGroup + Age + (1|DogID), data = EClec_
data)
summary(EC_mod)
```

```
## Cox mixed-effects model fit by maximum likelihood
##   Data: EClec_data
##   events, n = 1059, 1125
##   Iterations= 11 70
##                 NULL Integrated Fitted
## Log-likelihood -6570      -6150  -5925
##
##                     Chisq    df p    AIC   BIC
## Integrated loglik  839.3  13.0 0  813.3 748.8
##  Penalized loglik 1290.2 123.9 0 1042.4 427.2
##
## Model:  Surv(LEC, Event) ~ Trial + CI + BreedGroup + Age + (1 | DogID)
## Fixed coefficients
##                        coef exp(coef) se(coef)     z       p
## Trial2             0.009621    1.0097 0.138225  0.07 9.4e-01
## Trial3             0.193496    1.2135 0.135690  1.43 1.5e-01
## Trial4             0.400566    1.4927 0.135931  2.95 3.2e-03
## Trial5             0.589536    1.8032 0.137995  4.27 1.9e-05
## Trial6             0.665908    1.9463 0.137195  4.85 1.2e-06
## Trial7             0.426582    1.5320 0.138369  3.08 2.0e-03
## Trial8             0.612013    1.8441 0.137642  4.45 8.7e-06
## Trial9             0.610987    1.8422 0.140132  4.36 1.3e-05
## CI                 0.051220    1.0526 0.022198  2.31 2.1e-02
## BreedGroupMixed   -0.121918    0.8852 0.307611 -0.40 6.9e-01
## BreedGroupNoncoop -1.216281    0.2963 0.363931 -3.34 8.3e-04
## Age               -0.018497    0.9817 0.002727 -6.78 1.2e-11
```

```
##
## Random effects
##  Group Variable  Std Dev Variance
##  DogID Intercept 1.295   1.678
```

```
pairs(emmeans(EC_mod, ~Trial))
```

```
##  contrast estimate    SE  df z.ratio p.value
##  1 - 2     -0.0096 0.138 Inf -0.070  1.0000
##  1 - 3     -0.1935 0.136 Inf -1.426  0.8881
##  1 - 4     -0.4006 0.136 Inf -2.947  0.0780
##  1 - 5     -0.5895 0.138 Inf -4.272  0.0007
##  1 - 6     -0.6659 0.137 Inf -4.854  <.0001
##  1 - 7     -0.4266 0.138 Inf -3.083  0.0529
##  1 - 8     -0.6120 0.138 Inf -4.446  0.0003
##  1 - 9     -0.6110 0.140 Inf -4.360  0.0004
##  2 - 3     -0.1839 0.138 Inf -1.329  0.9229
##  2 - 4     -0.3909 0.138 Inf -2.828  0.1072
##  2 - 5     -0.5799 0.139 Inf -4.186  0.0009
##  2 - 6     -0.6563 0.140 Inf -4.693  0.0001
##  2 - 7     -0.4170 0.139 Inf -3.001  0.0671
##  2 - 8     -0.6024 0.141 Inf -4.274  0.0006
##  2 - 9     -0.6014 0.142 Inf -4.240  0.0008
##  3 - 4     -0.2071 0.138 Inf -1.504  0.8541
##  3 - 5     -0.3960 0.138 Inf -2.871  0.0958
##  3 - 6     -0.4724 0.138 Inf -3.411  0.0187
##  3 - 7     -0.2331 0.139 Inf -1.681  0.7587
##  3 - 8     -0.4185 0.139 Inf -3.006  0.0661
##  3 - 9     -0.4175 0.140 Inf -2.972  0.0727
##  4 - 5     -0.1890 0.137 Inf -1.375  0.9075
##  4 - 6     -0.2653 0.136 Inf -1.946  0.5813
##  4 - 7     -0.0260 0.137 Inf -0.190  1.0000
##  4 - 8     -0.2114 0.139 Inf -1.525  0.8443
##  4 - 9     -0.2104 0.140 Inf -1.503  0.8546
##  5 - 6     -0.0764 0.138 Inf -0.553  0.9998
##  5 - 7      0.1630 0.137 Inf  1.188  0.9592
##  5 - 8     -0.0225 0.137 Inf -0.164  1.0000
##  5 - 9     -0.0215 0.139 Inf -0.154  1.0000
##  6 - 7      0.2393 0.137 Inf  1.744  0.7191
##  6 - 8      0.0539 0.138 Inf  0.389  1.0000
##  6 - 9      0.0549 0.140 Inf  0.393  1.0000
##  7 - 8     -0.1854 0.139 Inf -1.333  0.9217
##  7 - 9     -0.1844 0.139 Inf -1.326  0.9240
##  8 - 9      0.0010 0.139 Inf  0.007  1.0000
##
## Results are averaged over the levels of: BreedGroup
## Results are given on the log (not the response) scale.
## P value adjustment: tukey method for comparing a family of 9 estimates
```

```
pairs(emmeans(EC_mod, ~BreedGroup))
```

```
##  contrast        estimate    SE  df z.ratio p.value
##  Coop - Mixed       0.122 0.308 Inf 0.396   0.9171
##  Coop - Noncoop     1.216 0.364 Inf 3.342   0.0024
##  Mixed - Noncoop    1.094 0.340 Inf 3.223   0.0036
##
## Results are averaged over the levels of: Trial
```

```
## Results are given on the log (not the response) scale.
## P value adjustment: tukey method for comparing a family of 3 estimates
```

j. 1-10. eye contact

```
EClec_data <- subset(EC_data, Trial==1 | Trial==2 | Trial==3 | Trial==4 | Trial==5 | Trial=
=6 | Trial==7 | Trial==8 | Trial==9 | Trial==10)
EClec_data$Trial <- factor(EClec_data$Trial,levels = c('1','2','3','4','5','6','7','8','9',
'10'))

EC_mod <- coxme(Surv(LEC, Event) ~ Trial + CI + BreedGroup + Age + (1|DogID), data = EClec_
data)
summary(EC_mod)

## Cox mixed-effects model fit by maximum likelihood
##   Data: EClec_data
##   events, n = 1172, 1250
##   Iterations= 18 107
##                   NULL Integrated Fitted
## Log-likelihood -7403      -6923  -6691
##
##                       Chisq    df p    AIC   BIC
## Integrated loglik  959.7  14.0 0  931.7 860.8
##  Penalized loglik 1424.8 125.2 0 1174.4 539.8
##
## Model:  Surv(LEC, Event) ~ Trial + CI + BreedGroup + Age + (1 | DogID)
## Fixed coefficients
##                          coef exp(coef) se(coef)     z       p
## Trial2                0.01177    1.0118 0.137997  0.09 9.3e-01
## Trial3                0.18950    1.2086 0.135636  1.40 1.6e-01
## Trial4                0.42413    1.5283 0.135467  3.13 1.7e-03
## Trial5                0.59310    1.8096 0.137765  4.31 1.7e-05
## Trial6                0.67662    1.9672 0.137082  4.94 8.0e-07
## Trial7                0.46253    1.5881 0.137909  3.35 8.0e-04
## Trial8                0.63416    1.8854 0.137382  4.62 3.9e-06
## Trial9                0.65742    1.9298 0.139688  4.71 2.5e-06
## Trial10               0.54698    1.7280 0.138857  3.94 8.2e-05
## CI                    0.05225    1.0536 0.022032  2.37 1.8e-02
## BreedGroupMixed      -0.10230    0.9028 0.305645 -0.33 7.4e-01
## BreedGroupNoncoop -1.16596    0.3116 0.357865 -3.26 1.1e-03
## Age                  -0.01802    0.9821 0.002651 -6.80 1.1e-11
##
## Random effects
##  Group Variable  Std Dev Variance
##  DogID Intercept 1.270   1.612

pairs(emmeans(EC_mod, ~Trial))

##  contrast estimate    SE  df z.ratio p.value
##  1 - 2     -0.0118 0.138 Inf -0.085  1.0000
##  1 - 3     -0.1895 0.136 Inf -1.397  0.9285
##  1 - 4     -0.4241 0.135 Inf -3.131  0.0552
##  1 - 5     -0.5931 0.138 Inf -4.305  0.0007
##  1 - 6     -0.6766 0.137 Inf -4.936  <.0001
##  1 - 7     -0.4625 0.138 Inf -3.354  0.0275
##  1 - 8     -0.6342 0.137 Inf -4.616  0.0002
##  1 - 9     -0.6574 0.140 Inf -4.706  0.0001
```

```
##   1 - 10    -0.5470 0.139 Inf -3.939  0.0033
##   2 - 3     -0.1777 0.138 Inf -1.289  0.9564
##   2 - 4     -0.4124 0.138 Inf -2.999  0.0806
##   2 - 5     -0.5813 0.138 Inf -4.212  0.0011
##   2 - 6     -0.6649 0.139 Inf -4.767  0.0001
##   2 - 7     -0.4508 0.138 Inf -3.263  0.0368
##   2 - 8     -0.6224 0.140 Inf -4.434  0.0004
##   2 - 9     -0.6457 0.141 Inf -4.578  0.0002
##   2 - 10    -0.5352 0.140 Inf -3.810  0.0054
##   3 - 4     -0.2346 0.137 Inf -1.714  0.7878
##   3 - 5     -0.4036 0.138 Inf -2.932  0.0967
##   3 - 6     -0.4871 0.138 Inf -3.524  0.0155
##   3 - 7     -0.2730 0.138 Inf -1.977  0.6151
##   3 - 8     -0.4447 0.139 Inf -3.205  0.0440
##   3 - 9     -0.4679 0.140 Inf -3.345  0.0283
##   3 - 10    -0.3575 0.140 Inf -2.559  0.2376
##   4 - 5     -0.1690 0.137 Inf -1.237  0.9665
##   4 - 6     -0.2525 0.136 Inf -1.860  0.6963
##   4 - 7     -0.0384 0.136 Inf -0.282  1.0000
##   4 - 8     -0.2100 0.138 Inf -1.526  0.8820
##   4 - 9     -0.2333 0.139 Inf -1.682  0.8059
##   4 - 10    -0.1228 0.137 Inf -0.897  0.9966
##   5 - 6     -0.0835 0.138 Inf -0.606  0.9999
##   5 - 7      0.1306 0.137 Inf  0.956  0.9945
##   5 - 8     -0.0411 0.137 Inf -0.300  1.0000
##   5 - 9     -0.0643 0.138 Inf -0.464  1.0000
##   5 - 10     0.0461 0.139 Inf  0.333  1.0000
##   6 - 7      0.2141 0.137 Inf  1.564  0.8657
##   6 - 8      0.0425 0.138 Inf  0.308  1.0000
##   6 - 9      0.0192 0.139 Inf  0.138  1.0000
##   6 - 10     0.1296 0.138 Inf  0.937  0.9953
##   7 - 8     -0.1716 0.138 Inf -1.242  0.9656
##   7 - 9     -0.1949 0.138 Inf -1.409  0.9249
##   7 - 10    -0.0845 0.137 Inf -0.618  0.9998
##   8 - 9     -0.0233 0.138 Inf -0.168  1.0000
##   8 - 10     0.0872 0.139 Inf  0.629  0.9998
##   9 - 10     0.1104 0.138 Inf  0.801  0.9986
## 
## Results are averaged over the levels of: BreedGroup 
## Results are given on the log (not the response) scale. 
## P value adjustment: tukey method for comparing a family of 10 estimates
```

```
pairs(emmeans(EC_mod, ~BreedGroup))
```

```
##  contrast        estimate    SE  df z.ratio p.value
##  Coop - Mixed       0.102 0.306 Inf 0.335   0.9401 
##  Coop - Noncoop     1.166 0.358 Inf 3.258   0.0032 
##  Mixed - Noncoop    1.064 0.336 Inf 3.169   0.0044 
## 
## Results are averaged over the levels of: Trial 
## Results are given on the log (not the response) scale. 
## P value adjustment: tukey method for comparing a family of 3 estimates
```

k. 1-11. eye contact

```
EClec_data <- subset(EC_data, Trial==1 | Trial==2 | Trial==3 | Trial==4 | Trial==5 | Trial=
=6 | Trial==7 | Trial==8 | Trial==9 | Trial==10 | Trial==11)
```

```
EClec_data$Trial <- factor(EClec_data$Trial,levels = c('1','2','3','4','5','6','7','8','9',
'10','11'))

EC_mod <- coxme(Surv(LEC, Event) ~ Trial + CI + BreedGroup + Age + (1|DogID), data = EClec_
data)
summary(EC_mod)

## Cox mixed-effects model fit by maximum likelihood
##   Data: EClec_data
##   events, n = 1283, 1375
##   Iterations= 31 173
##                    NULL Integrated Fitted
## Log-likelihood -8238      -7697  -7458
##
##                     Chisq    df p  AIC   BIC
## Integrated loglik  1083  15.0 0 1053 976.0
##  Penalized loglik  1560 126.6 0 1307 654.3
##
## Model:  Surv(LEC, Event) ~ Trial + CI + BreedGroup + Age + (1 | DogID)
## Fixed coefficients
##                         coef exp(coef) se(coef)     z       p
## Trial2               0.01915    1.0193 0.137502  0.14 8.9e-01
## Trial3               0.19278    1.2126 0.135272  1.43 1.5e-01
## Trial4               0.41213    1.5100 0.135162  3.05 2.3e-03
## Trial5               0.60895    1.8385 0.137271  4.44 9.2e-06
## Trial6               0.66246    1.9396 0.136691  4.85 1.3e-06
## Trial7               0.47108    1.6017 0.137409  3.43 6.1e-04
## Trial8               0.63619    1.8893 0.136870  4.65 3.3e-06
## Trial9               0.67263    1.9594 0.139136  4.83 1.3e-06
## Trial10              0.56707    1.7631 0.138445  4.10 4.2e-05
## Trial11              0.63375    1.8847 0.139023  4.56 5.1e-06
## CI                   0.05349    1.0549 0.021842  2.45 1.4e-02
## BreedGroupMixed     -0.09161    0.9125 0.300475 -0.30 7.6e-01
## BreedGroupNoncoop   -1.16761    0.3111 0.350524 -3.33 8.7e-04
## Age                 -0.01781    0.9823 0.002573 -6.92 4.5e-12
##
## Random effects
##  Group Variable  Std Dev Variance
##  DogID Intercept 1.247   1.554

pairs(emmeans(EC_mod, ~Trial))

##  contrast estimate    SE  df z.ratio p.value
##  1 - 2     -0.0192 0.138 Inf -0.139  1.0000
##  1 - 3     -0.1928 0.135 Inf -1.425  0.9424
##  1 - 4     -0.4121 0.135 Inf -3.049  0.0821
##  1 - 5     -0.6089 0.137 Inf -4.436  0.0005
##  1 - 6     -0.6625 0.137 Inf -4.846  0.0001
##  1 - 7     -0.4711 0.137 Inf -3.428  0.0256
##  1 - 8     -0.6362 0.137 Inf -4.648  0.0002
##  1 - 9     -0.6726 0.139 Inf -4.834  0.0001
##  1 - 10    -0.5671 0.138 Inf -4.096  0.0021
##  1 - 11    -0.6338 0.139 Inf -4.559  0.0003
##  2 - 3     -0.1736 0.137 Inf -1.264  0.9747
##  2 - 4     -0.3930 0.137 Inf -2.872  0.1319
##  2 - 5     -0.5898 0.138 Inf -4.289  0.0009
##  2 - 6     -0.6433 0.139 Inf -4.637  0.0002
```

```
##  2 - 7     -0.4519 0.137 Inf -3.288  0.0403
##  2 - 8     -0.6170 0.140 Inf -4.414  0.0005
##  2 - 9     -0.6535 0.140 Inf -4.657  0.0002
##  2 - 10    -0.5479 0.140 Inf -3.919  0.0043
##  2 - 11    -0.6146 0.141 Inf -4.371  0.0006
##  3 - 4     -0.2194 0.136 Inf -1.609  0.8789
##  3 - 5     -0.4162 0.137 Inf -3.035  0.0855
##  3 - 6     -0.4697 0.138 Inf -3.414  0.0269
##  3 - 7     -0.2783 0.137 Inf -2.025  0.6316
##  3 - 8     -0.4434 0.138 Inf -3.211  0.0511
##  3 - 9     -0.4799 0.139 Inf -3.445  0.0242
##  3 - 10    -0.3743 0.139 Inf -2.691  0.2038
##  3 - 11    -0.4410 0.140 Inf -3.160  0.0596
##  4 - 5     -0.1968 0.136 Inf -1.448  0.9362
##  4 - 6     -0.2503 0.135 Inf -1.848  0.7511
##  4 - 7     -0.0589 0.136 Inf -0.434  1.0000
##  4 - 8     -0.2241 0.137 Inf -1.633  0.8682
##  4 - 9     -0.2605 0.138 Inf -1.886  0.7268
##  4 - 10    -0.1549 0.136 Inf -1.136  0.9886
##  4 - 11    -0.2216 0.139 Inf -1.594  0.8852
##  5 - 6     -0.0535 0.137 Inf -0.390  1.0000
##  5 - 7      0.1379 0.136 Inf  1.014  0.9953
##  5 - 8     -0.0272 0.136 Inf -0.200  1.0000
##  5 - 9     -0.0637 0.138 Inf -0.462  1.0000
##  5 - 10     0.0419 0.138 Inf  0.303  1.0000
##  5 - 11    -0.0248 0.138 Inf -0.180  1.0000
##  6 - 7      0.1914 0.137 Inf  1.401  0.9485
##  6 - 8      0.0263 0.138 Inf  0.191  1.0000
##  6 - 9     -0.0102 0.139 Inf -0.073  1.0000
##  6 - 10     0.0954 0.138 Inf  0.692  0.9998
##  6 - 11     0.0287 0.140 Inf  0.205  1.0000
##  7 - 8     -0.1651 0.138 Inf -1.200  0.9827
##  7 - 9     -0.2016 0.138 Inf -1.463  0.9317
##  7 - 10    -0.0960 0.136 Inf -0.704  0.9998
##  7 - 11    -0.1627 0.138 Inf -1.176  0.9851
##  8 - 9     -0.0364 0.138 Inf -0.264  1.0000
##  8 - 10     0.0691 0.138 Inf  0.500  1.0000
##  8 - 11     0.0024 0.139 Inf  0.018  1.0000
##  9 - 10     0.1056 0.138 Inf  0.767  0.9996
##  9 - 11     0.0389 0.139 Inf  0.279  1.0000
##  10 - 11   -0.0667 0.139 Inf -0.480  1.0000
##
## Results are averaged over the levels of: BreedGroup
## Results are given on the log (not the response) scale.
## P value adjustment: tukey method for comparing a family of 11 estimates
```

```
pairs(emmeans(EC_mod, ~BreedGroup))
```

```
##  contrast        estimate    SE  df z.ratio p.value
##  Coop - Mixed      0.0916 0.300 Inf 0.305   0.9501
##  Coop - Noncoop    1.1676 0.350 Inf 3.331   0.0025
##  Mixed - Noncoop   1.0760 0.327 Inf 3.293   0.0029
##
## Results are averaged over the levels of: Trial
## Results are given on the log (not the response) scale.
## P value adjustment: tukey method for comparing a family of 3 estimates
```

l. 1-12. eye contact

```
EClec_data <- subset(EC_data, Trial==1 | Trial==2 | Trial==3 | Trial==4 | Trial==5 | Trial=
=6 | Trial==7 | Trial==8 | Trial==9 | Trial==10 | Trial==11 | Trial==12)
EClec_data$Trial <- factor(EClec_data$Trial,levels = c('1','2','3','4','5','6','7','8','9',
'10','11','12'))

EC_mod <- coxme(Surv(LEC, Event) ~ Trial + CI + BreedGroup + Age + Play + (1|DogID), data =
EClec_data)
summary(EC_mod)

## Cox mixed-effects model fit by maximum likelihood
##   Data: EClec_data
##   events, n = 1394, 1500
##   Iterations= 21 122
##                   NULL Integrated Fitted
## Log-likelihood -9083      -8471  -8229
##
##                     Chisq    df p  AIC    BIC
## Integrated loglik  1223  17.0 0 1189 1100.4
##  Penalized loglik  1707 126.5 0 1454  791.8
##
## Model:  Surv(LEC, Event) ~ Trial + CI + BreedGroup + Age + Play + (1 |      DogID)
## Fixed coefficients
##                         coef exp(coef) se(coef)     z       p
## Trial2              0.01471    1.0148 0.136660  0.11 9.1e-01
## Trial3              0.18125    1.1987 0.134909  1.34 1.8e-01
## Trial4              0.40718    1.5026 0.134495  3.03 2.5e-03
## Trial5              0.60220    1.8261 0.136653  4.41 1.0e-05
## Trial6              0.64282    1.9018 0.136004  4.73 2.3e-06
## Trial7              0.46744    1.5959 0.136688  3.42 6.3e-04
## Trial8              0.64183    1.8999 0.136301  4.71 2.5e-06
## Trial9              0.68133    1.9765 0.138561  4.92 8.8e-07
## Trial10             0.58346    1.7922 0.137827  4.23 2.3e-05
## Trial11             0.65331    1.9219 0.138169  4.73 2.3e-06
## Trial12             0.70416    2.0221 0.137889  5.11 3.3e-07
## CI                  0.05306    1.0545 0.019310  2.75 6.0e-03
## BreedGroupMixed    -0.02110    0.9791 0.266151 -0.08 9.4e-01
## BreedGroupNoncoop -1.10280    0.3319 0.314956 -3.50 4.6e-04
## Age                -0.01530    0.9848 0.002812 -5.44 5.3e-08
## Play1               0.49251    1.6364 0.241195  2.04 4.1e-02
##
## Random effects
##  Group Variable  Std Dev Variance
##  DogID Intercept 1.114   1.242

pairs(emmeans(EC_mod, ~Trial))

##  contrast estimate    SE  df z.ratio p.value
##  1 - 2     -0.0147 0.137 Inf -0.108  1.0000
##  1 - 3     -0.1812 0.135 Inf -1.343  0.9734
##  1 - 4     -0.4072 0.134 Inf -3.027  0.1006
##  1 - 5     -0.6022 0.137 Inf -4.407  0.0006
##  1 - 6     -0.6428 0.136 Inf -4.726  0.0001
##  1 - 7     -0.4674 0.137 Inf -3.420  0.0308
##  1 - 8     -0.6418 0.136 Inf -4.709  0.0002
##  1 - 9     -0.6813 0.139 Inf -4.917  0.0001
```

```
##  1 - 10    -0.5835 0.138 Inf -4.233  0.0014
##  1 - 11    -0.6533 0.138 Inf -4.728  0.0001
##  1 - 12    -0.7042 0.138 Inf -5.107  <.0001
##  2 - 3     -0.1665 0.137 Inf -1.219  0.9876
##  2 - 4     -0.3925 0.136 Inf -2.885  0.1462
##  2 - 5     -0.5875 0.137 Inf -4.296  0.0011
##  2 - 6     -0.6281 0.138 Inf -4.550  0.0003
##  2 - 7     -0.4527 0.137 Inf -3.310  0.0438
##  2 - 8     -0.6271 0.139 Inf -4.514  0.0004
##  2 - 9     -0.6666 0.140 Inf -4.774  0.0001
##  2 - 10    -0.5687 0.139 Inf -4.092  0.0025
##  2 - 11    -0.6386 0.140 Inf -4.567  0.0003
##  2 - 12    -0.6895 0.140 Inf -4.930  0.0001
##  3 - 4     -0.2259 0.136 Inf -1.666  0.8836
##  3 - 5     -0.4210 0.137 Inf -3.080  0.0871
##  3 - 6     -0.4616 0.137 Inf -3.369  0.0364
##  3 - 7     -0.2862 0.137 Inf -2.091  0.6292
##  3 - 8     -0.4606 0.137 Inf -3.353  0.0383
##  3 - 9     -0.5001 0.139 Inf -3.603  0.0165
##  3 - 10    -0.4022 0.139 Inf -2.903  0.1397
##  3 - 11    -0.4721 0.139 Inf -3.401  0.0327
##  3 - 12    -0.5229 0.139 Inf -3.761  0.0093
##  4 - 5     -0.1950 0.135 Inf -1.441  0.9555
##  4 - 6     -0.2356 0.135 Inf -1.745  0.8471
##  4 - 7     -0.0603 0.135 Inf -0.445  1.0000
##  4 - 8     -0.2346 0.136 Inf -1.719  0.8598
##  4 - 9     -0.2742 0.138 Inf -1.992  0.6993
##  4 - 10    -0.1763 0.136 Inf -1.296  0.9798
##  4 - 11    -0.2461 0.138 Inf -1.782  0.8284
##  4 - 12    -0.2970 0.137 Inf -2.166  0.5746
##  5 - 6     -0.0406 0.137 Inf -0.297  1.0000
##  5 - 7      0.1348 0.136 Inf  0.995  0.9978
##  5 - 8     -0.0396 0.136 Inf -0.291  1.0000
##  5 - 9     -0.0791 0.138 Inf -0.575  1.0000
##  5 - 10     0.0187 0.137 Inf  0.136  1.0000
##  5 - 11    -0.0511 0.138 Inf -0.371  1.0000
##  5 - 12    -0.1020 0.138 Inf -0.741  0.9999
##  6 - 7      0.1754 0.136 Inf  1.287  0.9808
##  6 - 8      0.0010 0.137 Inf  0.007  1.0000
##  6 - 9     -0.0385 0.138 Inf -0.278  1.0000
##  6 - 10     0.0594 0.138 Inf  0.432  1.0000
##  6 - 11    -0.0105 0.139 Inf -0.075  1.0000
##  6 - 12    -0.0613 0.139 Inf -0.442  1.0000
##  7 - 8     -0.1744 0.137 Inf -1.273  0.9825
##  7 - 9     -0.2139 0.137 Inf -1.558  0.9240
##  7 - 10    -0.1160 0.136 Inf -0.854  0.9995
##  7 - 11    -0.1859 0.138 Inf -1.352  0.9722
##  7 - 12    -0.2367 0.137 Inf -1.724  0.8576
##  8 - 9     -0.0395 0.137 Inf -0.287  1.0000
##  8 - 10     0.0584 0.138 Inf  0.424  1.0000
##  8 - 11    -0.0115 0.139 Inf -0.083  1.0000
##  8 - 12    -0.0623 0.137 Inf -0.453  1.0000
##  9 - 10     0.0979 0.137 Inf  0.712  0.9999
##  9 - 11     0.0280 0.139 Inf  0.202  1.0000
##  9 - 12    -0.0228 0.138 Inf -0.165  1.0000
##  10 - 11   -0.0699 0.138 Inf -0.505  1.0000
##  10 - 12   -0.1207 0.137 Inf -0.879  0.9993
```

```
##  11 - 12   -0.0508 0.138 Inf -0.368  1.0000
##
## Results are averaged over the levels of: BreedGroup, Play
## Results are given on the log (not the response) scale.
## P value adjustment: tukey method for comparing a family of 12 estimates
```

```
pairs(emmeans(EC_mod, ~BreedGroup))
```

```
##  contrast        estimate    SE  df z.ratio p.value
##  Coop - Mixed      0.0211 0.266 Inf 0.079   0.9965
##  Coop - Noncoop    1.1028 0.315 Inf 3.501   0.0013
##  Mixed - Noncoop   1.0817 0.291 Inf 3.716   0.0006
##
## Results are averaged over the levels of: Trial, Play
## Results are given on the log (not the response) scale.
## P value adjustment: tukey method for comparing a family of 3 estimates
```

m. 1-13. eye contact

```
EClec_data <- subset(EC_data, Trial==1 | Trial==2 | Trial==3 | Trial==4 | Trial==5 | Trial=
=6 | Trial==7 | Trial==8 | Trial==9 | Trial==10 | Trial==11 | Trial==12 | Trial==13)
EClec_data$Trial <- factor(EClec_data$Trial,levels = c('1','2','3','4','5','6','7','8','9',
'10','11','12','13'))

EC_mod <- coxme(Surv(LEC, Event) ~ Trial + CI + BreedGroup + Age + Play + (1|DogID), data =
EClec_data)
summary(EC_mod)
```

```
## Cox mixed-effects model fit by maximum likelihood
##   Data: EClec_data
##   events, n = 1504, 1625
##   Iterations= 21 122
##                   NULL Integrated Fitted
## Log-likelihood -9931      -9262  -9014
##
##                     Chisq    df p  AIC    BIC
## Integrated loglik  1338  18.0 0 1302 1206.2
##  Penalized loglik  1833 127.5 0 1578  900.5
##
## Model:  Surv(LEC, Event) ~ Trial + CI + BreedGroup + Age + Play + (1 |      DogID)
## Fixed coefficients
##                         coef exp(coef) se(coef)     z       p
## Trial2               0.04678    1.0479 0.136182  0.34 7.3e-01
## Trial3               0.18982    1.2090 0.134475  1.41 1.6e-01
## Trial4               0.42100    1.5235 0.134228  3.14 1.7e-03
## Trial5               0.60780    1.8364 0.136310  4.46 8.2e-06
## Trial6               0.65342    1.9221 0.135705  4.81 1.5e-06
## Trial7               0.48815    1.6293 0.136256  3.58 3.4e-04
## Trial8               0.66010    1.9350 0.136013  4.85 1.2e-06
## Trial9               0.70078    2.0153 0.138256  5.07 4.0e-07
## Trial10              0.61151    1.8432 0.137444  4.45 8.6e-06
## Trial11              0.67668    1.9673 0.137806  4.91 9.1e-07
## Trial12              0.73653    2.0887 0.137540  5.36 8.6e-08
## Trial13              0.81392    2.2567 0.140011  5.81 6.1e-09
## CI                   0.05191    1.0533 0.018687  2.78 5.5e-03
## BreedGroupMixed     -0.03651    0.9641 0.256298 -0.14 8.9e-01
## BreedGroupNoncoop -1.10516    0.3312 0.303314 -3.64 2.7e-04
```

```
## Age              -0.01480    0.9853 0.002739 -5.40 6.5e-08
## Play1             0.48562    1.6252 0.232914  2.08 3.7e-02
##
## Random effects
##  Group Variable  Std Dev Variance
##  DogID Intercept 1.076   1.159
```

```
pairs(emmeans(EC_mod, ~Trial))
```

```
##  contrast estimate    SE  df z.ratio p.value
##  1 - 2     -0.0468 0.136 Inf -0.344  1.0000
##  1 - 3     -0.1898 0.134 Inf -1.412  0.9728
##  1 - 4     -0.4210 0.134 Inf -3.136  0.0846
##  1 - 5     -0.6078 0.136 Inf -4.459  0.0006
##  1 - 6     -0.6534 0.136 Inf -4.815  0.0001
##  1 - 7     -0.4881 0.136 Inf -3.583  0.0205
##  1 - 8     -0.6601 0.136 Inf -4.853  0.0001
##  1 - 9     -0.7008 0.138 Inf -5.069  <.0001
##  1 - 10    -0.6115 0.137 Inf -4.449  0.0006
##  1 - 11    -0.6767 0.138 Inf -4.910  0.0001
##  1 - 12    -0.7365 0.138 Inf -5.355  <.0001
##  1 - 13    -0.8139 0.140 Inf -5.813  <.0001
##  2 - 3     -0.1430 0.136 Inf -1.054  0.9980
##  2 - 4     -0.3742 0.135 Inf -2.763  0.2196
##  2 - 5     -0.5610 0.136 Inf -4.123  0.0026
##  2 - 6     -0.6066 0.137 Inf -4.419  0.0007
##  2 - 7     -0.4414 0.136 Inf -3.241  0.0622
##  2 - 8     -0.6133 0.138 Inf -4.440  0.0007
##  2 - 9     -0.6540 0.139 Inf -4.701  0.0002
##  2 - 10    -0.5647 0.138 Inf -4.089  0.0030
##  2 - 11    -0.6299 0.139 Inf -4.528  0.0004
##  2 - 12    -0.6897 0.139 Inf -4.956  0.0001
##  2 - 13    -0.7671 0.140 Inf -5.492  <.0001
##  3 - 4     -0.2312 0.135 Inf -1.714  0.8896
##  3 - 5     -0.4180 0.136 Inf -3.074  0.1008
##  3 - 6     -0.4636 0.137 Inf -3.395  0.0385
##  3 - 7     -0.2983 0.136 Inf -2.192  0.5968
##  3 - 8     -0.4703 0.137 Inf -3.440  0.0332
##  3 - 9     -0.5110 0.138 Inf -3.699  0.0136
##  3 - 10    -0.4217 0.138 Inf -3.061  0.1043
##  3 - 11    -0.4869 0.138 Inf -3.529  0.0247
##  3 - 12    -0.5467 0.138 Inf -3.954  0.0051
##  3 - 13    -0.6241 0.140 Inf -4.465  0.0006
##  4 - 5     -0.1868 0.135 Inf -1.385  0.9767
##  4 - 6     -0.2324 0.135 Inf -1.726  0.8844
##  4 - 7     -0.0672 0.135 Inf -0.498  1.0000
##  4 - 8     -0.2391 0.136 Inf -1.759  0.8700
##  4 - 9     -0.2798 0.137 Inf -2.039  0.7067
##  4 - 10    -0.1905 0.136 Inf -1.404  0.9740
##  4 - 11    -0.2557 0.138 Inf -1.859  0.8193
##  4 - 12    -0.3155 0.137 Inf -2.310  0.5092
##  4 - 13    -0.3929 0.138 Inf -2.845  0.1817
##  5 - 6     -0.0456 0.136 Inf -0.334  1.0000
##  5 - 7      0.1197 0.135 Inf  0.886  0.9996
##  5 - 8     -0.0523 0.136 Inf -0.386  1.0000
##  5 - 9     -0.0930 0.137 Inf -0.678  1.0000
##  5 - 10    -0.0037 0.137 Inf -0.027  1.0000
```

```
##  5 - 11    -0.0689 0.137 Inf -0.502  1.0000
##  5 - 12    -0.1287 0.137 Inf -0.938  0.9994
##  5 - 13    -0.2061 0.139 Inf -1.483  0.9601
##  6 - 7      0.1653 0.136 Inf  1.218  0.9922
##  6 - 8     -0.0067 0.137 Inf -0.049  1.0000
##  6 - 9     -0.0474 0.138 Inf -0.343  1.0000
##  6 - 10     0.0419 0.137 Inf  0.306  1.0000
##  6 - 11    -0.0233 0.138 Inf -0.168  1.0000
##  6 - 12    -0.0831 0.138 Inf -0.602  1.0000
##  6 - 13    -0.1605 0.139 Inf -1.156  0.9951
##  7 - 8     -0.1720 0.136 Inf -1.261  0.9894
##  7 - 9     -0.2126 0.137 Inf -1.553  0.9437
##  7 - 10    -0.1234 0.135 Inf -0.911  0.9995
##  7 - 11    -0.1885 0.137 Inf -1.377  0.9777
##  7 - 12    -0.2484 0.137 Inf -1.816  0.8423
##  7 - 13    -0.3258 0.138 Inf -2.361  0.4718
##  8 - 9     -0.0407 0.137 Inf -0.297  1.0000
##  8 - 10     0.0486 0.137 Inf  0.354  1.0000
##  8 - 11    -0.0166 0.138 Inf -0.120  1.0000
##  8 - 12    -0.0764 0.137 Inf -0.558  1.0000
##  8 - 13    -0.1538 0.139 Inf -1.109  0.9967
##  9 - 10     0.0893 0.137 Inf  0.651  1.0000
##  9 - 11     0.0241 0.138 Inf  0.174  1.0000
##  9 - 12    -0.0357 0.138 Inf -0.259  1.0000
##  9 - 13    -0.1131 0.139 Inf -0.811  0.9999
##  10 - 11   -0.0652 0.138 Inf -0.473  1.0000
##  10 - 12   -0.1250 0.137 Inf -0.913  0.9995
##  10 - 13   -0.2024 0.138 Inf -1.463  0.9641
##  11 - 12   -0.0598 0.138 Inf -0.433  1.0000
##  11 - 13   -0.1372 0.140 Inf -0.981  0.9990
##  12 - 13   -0.0774 0.139 Inf -0.558  1.0000
##
## Results are averaged over the levels of: BreedGroup, Play
## Results are given on the log (not the response) scale.
## P value adjustment: tukey method for comparing a family of 13 estimates
```

```
pairs(emmeans(EC_mod, ~BreedGroup))
```

```
##  contrast        estimate    SE  df z.ratio p.value
##  Coop - Mixed      0.0365 0.256 Inf 0.142   0.9889
##  Coop - Noncoop    1.1052 0.303 Inf 3.644   0.0008
##  Mixed - Noncoop   1.0687 0.279 Inf 3.830   0.0004
##
## Results are averaged over the levels of: Trial, Play
## Results are given on the log (not the response) scale.
## P value adjustment: tukey method for comparing a family of 3 estimates
```

n. 1-14. eye contact

```
EClec_data <- subset(EC_data, Trial==1 | Trial==2 | Trial==3 | Trial==4 | Trial==5 | Trial=
=6 | Trial==7 | Trial==8 | Trial==9 | Trial==10 | Trial==11 | Trial==12 | Trial==13 | Trial
==14)
EClec_data$Trial <- factor(EClec_data$Trial,levels = c('1','2','3','4','5','6','7','8','9',
'10','11','12','13','14'))

EC_mod <- coxme(Surv(LEC, Event) ~ Trial + CI + BreedGroup + Age + Play + (1|DogID), data =
```

```
EClec_data)
summary(EC_mod)

## Cox mixed-effects model fit by maximum likelihood
##   Data: EClec_data
##   events, n = 1613, 1750
##   Iterations= 32 178
##                   NULL Integrated Fitted
## Log-likelihood -10782     -10053  -9800
##
##                    Chisq    df p  AIC  BIC
## Integrated loglik  1458  19.0 0 1420 1318
##  Penalized loglik  1964 129.1 0 1706 1010
##
## Model:  Surv(LEC, Event) ~ Trial + CI + BreedGroup + Age + Play + (1 |      DogID)
## Fixed coefficients
##                         coef exp(coef) se(coef)     z       p
## Trial2              0.04834    1.0495 0.135584  0.36 7.2e-01
## Trial3              0.17542    1.1917 0.134018  1.31 1.9e-01
## Trial4              0.42409    1.5282 0.133783  3.17 1.5e-03
## Trial5              0.61352    1.8469 0.135822  4.52 6.3e-06
## Trial6              0.64984    1.9152 0.135153  4.81 1.5e-06
## Trial7              0.48160    1.6187 0.135746  3.55 3.9e-04
## Trial8              0.65581    1.9267 0.135390  4.84 1.3e-06
## Trial9              0.70298    2.0198 0.137676  5.11 3.3e-07
## Trial10             0.61822    1.8556 0.136907  4.52 6.3e-06
## Trial11             0.68609    1.9859 0.137467  4.99 6.0e-07
## Trial12             0.76302    2.1447 0.136944  5.57 2.5e-08
## Trial13             0.83372    2.3019 0.139447  5.98 2.2e-09
## Trial14             0.55735    1.7460 0.137795  4.04 5.2e-05
## CI                  0.05326    1.0547 0.019097  2.79 5.3e-03
## BreedGroupMixed    -0.01351    0.9866 0.263195 -0.05 9.6e-01
## BreedGroupNoncoop -1.08905    0.3365 0.310994 -3.50 4.6e-04
## Age                -0.01454    0.9856 0.002803 -5.19 2.1e-07
## Play1               0.50344    1.6544 0.236800  2.13 3.4e-02
##
## Random effects
##  Group Variable  Std Dev Variance
##  DogID Intercept 1.094   1.197

pairs(emmeans(EC_mod, ~Trial))

##  contrast estimate    SE  df z.ratio p.value
##  1 - 2     -0.0483 0.136 Inf -0.357  1.0000
##  1 - 3     -0.1754 0.134 Inf -1.309  0.9902
##  1 - 4     -0.4241 0.134 Inf -3.170  0.0868
##  1 - 5     -0.6135 0.136 Inf -4.517  0.0005
##  1 - 6     -0.6498 0.135 Inf -4.808  0.0001
##  1 - 7     -0.4816 0.136 Inf -3.548  0.0265
##  1 - 8     -0.6558 0.135 Inf -4.844  0.0001
##  1 - 9     -0.7030 0.138 Inf -5.106  <.0001
##  1 - 10    -0.6182 0.137 Inf -4.516  0.0005
##  1 - 11    -0.6861 0.137 Inf -4.991  0.0001
##  1 - 12    -0.7630 0.137 Inf -5.572  <.0001
##  1 - 13    -0.8337 0.139 Inf -5.979  <.0001
##  1 - 14    -0.5574 0.138 Inf -4.045  0.0041
##  2 - 3     -0.1271 0.135 Inf -0.938  0.9997
```

```
##  2 - 4     -0.3758 0.135 Inf -2.781  0.2341
##  2 - 5     -0.5652 0.136 Inf -4.165  0.0025
##  2 - 6     -0.6015 0.137 Inf -4.395  0.0009
##  2 - 7     -0.4333 0.136 Inf -3.191  0.0817
##  2 - 8     -0.6075 0.138 Inf -4.413  0.0009
##  2 - 9     -0.6546 0.139 Inf -4.721  0.0002
##  2 - 10    -0.5699 0.138 Inf -4.139  0.0028
##  2 - 11    -0.6378 0.139 Inf -4.595  0.0004
##  2 - 12    -0.7147 0.139 Inf -5.157  <.0001
##  2 - 13    -0.7854 0.139 Inf -5.636  <.0001
##  2 - 14    -0.5090 0.139 Inf -3.664  0.0176
##  3 - 4     -0.2487 0.135 Inf -1.846  0.8549
##  3 - 5     -0.4381 0.136 Inf -3.227  0.0736
##  3 - 6     -0.4744 0.136 Inf -3.480  0.0333
##  3 - 7     -0.3062 0.136 Inf -2.253  0.5902
##  3 - 8     -0.4804 0.136 Inf -3.521  0.0290
##  3 - 9     -0.5276 0.138 Inf -3.826  0.0097
##  3 - 10    -0.4428 0.138 Inf -3.220  0.0751
##  3 - 11    -0.5107 0.138 Inf -3.707  0.0151
##  3 - 12    -0.5876 0.138 Inf -4.260  0.0017
##  3 - 13    -0.6583 0.140 Inf -4.718  0.0002
##  3 - 14    -0.3819 0.139 Inf -2.742  0.2551
##  4 - 5     -0.1894 0.135 Inf -1.408  0.9813
##  4 - 6     -0.2258 0.135 Inf -1.677  0.9242
##  4 - 7     -0.0575 0.135 Inf -0.427  1.0000
##  4 - 8     -0.2317 0.136 Inf -1.708  0.9135
##  4 - 9     -0.2789 0.137 Inf -2.036  0.7453
##  4 - 10    -0.1941 0.135 Inf -1.433  0.9782
##  4 - 11    -0.2620 0.137 Inf -1.907  0.8231
##  4 - 12    -0.3389 0.136 Inf -2.487  0.4172
##  4 - 13    -0.4096 0.138 Inf -2.974  0.1476
##  4 - 14    -0.1333 0.137 Inf -0.972  0.9995
##  5 - 6     -0.0363 0.136 Inf -0.267  1.0000
##  5 - 7      0.1319 0.135 Inf  0.978  0.9995
##  5 - 8     -0.0423 0.135 Inf -0.312  1.0000
##  5 - 9     -0.0895 0.137 Inf -0.654  1.0000
##  5 - 10    -0.0047 0.137 Inf -0.034  1.0000
##  5 - 11    -0.0726 0.137 Inf -0.529  1.0000
##  5 - 12    -0.1495 0.137 Inf -1.092  0.9983
##  5 - 13    -0.2202 0.139 Inf -1.588  0.9496
##  5 - 14     0.0562 0.138 Inf  0.408  1.0000
##  6 - 7      0.1682 0.136 Inf  1.241  0.9941
##  6 - 8     -0.0060 0.136 Inf -0.044  1.0000
##  6 - 9     -0.0531 0.138 Inf -0.385  1.0000
##  6 - 10     0.0316 0.137 Inf  0.231  1.0000
##  6 - 11    -0.0363 0.138 Inf -0.262  1.0000
##  6 - 12    -0.1132 0.138 Inf -0.822  0.9999
##  6 - 13    -0.1839 0.139 Inf -1.327  0.9889
##  6 - 14     0.0925 0.138 Inf  0.668  1.0000
##  7 - 8     -0.1742 0.136 Inf -1.280  0.9921
##  7 - 9     -0.2214 0.137 Inf -1.620  0.9412
##  7 - 10    -0.1366 0.135 Inf -1.010  0.9993
##  7 - 11    -0.2045 0.137 Inf -1.496  0.9687
##  7 - 12    -0.2814 0.136 Inf -2.062  0.7278
##  7 - 13    -0.3521 0.138 Inf -2.558  0.3680
##  7 - 14    -0.0758 0.138 Inf -0.548  1.0000
##  8 - 9     -0.0472 0.137 Inf -0.344  1.0000
```

```
##  8 - 10     0.0376 0.137 Inf  0.275  1.0000
##  8 - 11    -0.0303 0.138 Inf -0.219  1.0000
##  8 - 12    -0.1072 0.137 Inf -0.785  1.0000
##  8 - 13    -0.1779 0.138 Inf -1.285  0.9918
##  8 - 14     0.0985 0.139 Inf  0.711  1.0000
##  9 - 10     0.0848 0.137 Inf  0.619  1.0000
##  9 - 11     0.0169 0.138 Inf  0.122  1.0000
##  9 - 12    -0.0600 0.138 Inf -0.436  1.0000
##  9 - 13    -0.1307 0.139 Inf -0.939  0.9997
##  9 - 14     0.1456 0.139 Inf  1.046  0.9989
##  10 - 11   -0.0679 0.138 Inf -0.493  1.0000
##  10 - 12   -0.1448 0.137 Inf -1.059  0.9988
##  10 - 13   -0.2155 0.138 Inf -1.560  0.9561
##  10 - 14    0.0609 0.138 Inf  0.440  1.0000
##  11 - 12   -0.0769 0.138 Inf -0.557  1.0000
##  11 - 13   -0.1476 0.140 Inf -1.055  0.9988
##  11 - 14    0.1287 0.140 Inf  0.922  0.9997
##  12 - 13   -0.0707 0.139 Inf -0.510  1.0000
##  12 - 14    0.2057 0.138 Inf  1.492  0.9694
##  13 - 14    0.2764 0.140 Inf  1.978  0.7817
##
## Results are averaged over the levels of: BreedGroup, Play
## Results are given on the log (not the response) scale.
## P value adjustment: tukey method for comparing a family of 14 estimates

pairs(emmeans(EC_mod, ~BreedGroup))

##  contrast        estimate    SE  df z.ratio p.value
##  Coop - Mixed      0.0135 0.263 Inf 0.051   0.9985
##  Coop - Noncoop    1.0890 0.311 Inf 3.502   0.0013
##  Mixed - Noncoop   1.0755 0.285 Inf 3.775   0.0005
##
## Results are averaged over the levels of: Trial, Play
## Results are given on the log (not the response) scale.
## P value adjustment: tukey method for comparing a family of 3 estimates
```

o. 1-15. eye contact

```
EClec_data <- subset(EC_data, Trial==1 | Trial==2 | Trial==3 | Trial==4 | Trial==5 | Trial=
=6 | Trial==7 | Trial==8 | Trial==9 | Trial==10 | Trial==11 | Trial==12 | Trial==13 | Trial
==14 | Trial==15)
EClec_data$Trial <- factor(EClec_data$Trial,levels = c('1','2','3','4','5','6','7','8','9',
'10','11','12','13','14','15'))

EC_mod <- coxme(Surv(LEC, Event) ~ Trial + CI + BreedGroup + Age + Play + (1|DogID), data =
EClec_data)
summary(EC_mod)

## Cox mixed-effects model fit by maximum likelihood
##   Data: EClec_data
##   events, n = 1722, 1875
##   Iterations= 15 91
##                   NULL Integrated Fitted
## Log-likelihood -11640     -10852 -10594
##
##                   Chisq  df p  AIC  BIC
## Integrated loglik  1576  20 0 1536 1427
```

```
##  Penalized loglik  2093 130 0 1833 1124
##
## Model:  Surv(LEC, Event) ~ Trial + CI + BreedGroup + Age + Play + (1 |      DogID)
## Fixed coefficients
##                          coef exp(coef) se(coef)     z       p
## Trial2                0.05155    1.0529 0.135161  0.38 7.0e-01
## Trial3                0.18101    1.1984 0.133714  1.35 1.8e-01
## Trial4                0.42467    1.5291 0.133549  3.18 1.5e-03
## Trial5                0.62033    1.8595 0.135456  4.58 4.7e-06
## Trial6                0.64176    1.8998 0.134994  4.75 2.0e-06
## Trial7                0.48484    1.6239 0.135408  3.58 3.4e-04
## Trial8                0.65621    1.9275 0.135072  4.86 1.2e-06
## Trial9                0.69865    2.0110 0.137392  5.09 3.7e-07
## Trial10               0.63043    1.8784 0.136596  4.62 3.9e-06
## Trial11               0.70013    2.0140 0.137207  5.10 3.3e-07
## Trial12               0.77089    2.1617 0.136591  5.64 1.7e-08
## Trial13               0.84302    2.3234 0.139037  6.06 1.3e-09
## Trial14               0.57369    1.7748 0.137465  4.17 3.0e-05
## Trial15               0.72470    2.0641 0.138162  5.25 1.6e-07
## CI                    0.05381    1.0553 0.018295  2.94 3.3e-03
## BreedGroupMixed      -0.01015    0.9899 0.251391 -0.04 9.7e-01
## BreedGroupNoncoop    -1.08678    0.3373 0.298336 -3.64 2.7e-04
## Age                  -0.01428    0.9858 0.002707 -5.27 1.3e-07
## Play1                 0.51487    1.6734 0.226305  2.28 2.3e-02
##
## Random effects
##  Group Variable  Std Dev Variance
##  DogID Intercept 1.051   1.106
```

```
pairs(emmeans(EC_mod, ~Trial))
```

```
##  contrast estimate    SE  df z.ratio p.value
##  1 - 2     -0.0515 0.135 Inf -0.381  1.0000
##  1 - 3     -0.1810 0.134 Inf -1.354  0.9909
##  1 - 4     -0.4247 0.134 Inf -3.180  0.0943
##  1 - 5     -0.6203 0.135 Inf -4.580  0.0005
##  1 - 6     -0.6418 0.135 Inf -4.754  0.0002
##  1 - 7     -0.4848 0.135 Inf -3.581  0.0268
##  1 - 8     -0.6562 0.135 Inf -4.858  0.0001
##  1 - 9     -0.6987 0.137 Inf -5.085  <.0001
##  1 - 10    -0.6304 0.137 Inf -4.615  0.0004
##  1 - 11    -0.7001 0.137 Inf -5.103  <.0001
##  1 - 12    -0.7709 0.137 Inf -5.644  <.0001
##  1 - 13    -0.8430 0.139 Inf -6.063  <.0001
##  1 - 14    -0.5737 0.137 Inf -4.173  0.0028
##  1 - 15    -0.7247 0.138 Inf -5.245  <.0001
##  2 - 3     -0.1295 0.135 Inf -0.960  0.9998
##  2 - 4     -0.3731 0.135 Inf -2.769  0.2636
##  2 - 5     -0.5688 0.135 Inf -4.207  0.0024
##  2 - 6     -0.5902 0.136 Inf -4.325  0.0014
##  2 - 7     -0.4333 0.135 Inf -3.202  0.0887
##  2 - 8     -0.6047 0.137 Inf -4.407  0.0010
##  2 - 9     -0.6471 0.138 Inf -4.682  0.0003
##  2 - 10    -0.5789 0.137 Inf -4.220  0.0023
##  2 - 11    -0.6486 0.138 Inf -4.689  0.0003
##  2 - 12    -0.7193 0.138 Inf -5.211  <.0001
##  2 - 13    -0.7915 0.139 Inf -5.700  <.0001
```

```
##  2 - 14    -0.5221 0.138 Inf -3.772  0.0135
##  2 - 15    -0.6732 0.139 Inf -4.839  0.0001
##  3 - 4     -0.2437 0.134 Inf -1.815  0.8927
##  3 - 5     -0.4393 0.135 Inf -3.249  0.0773
##  3 - 6     -0.4608 0.136 Inf -3.390  0.0502
##  3 - 7     -0.3038 0.135 Inf -2.244  0.6324
##  3 - 8     -0.4752 0.136 Inf -3.494  0.0358
##  3 - 9     -0.5176 0.137 Inf -3.769  0.0137
##  3 - 10    -0.4494 0.137 Inf -3.283  0.0698
##  3 - 11    -0.5191 0.137 Inf -3.783  0.0130
##  3 - 12    -0.5899 0.137 Inf -4.293  0.0017
##  3 - 13    -0.6620 0.139 Inf -4.764  0.0002
##  3 - 14    -0.3927 0.139 Inf -2.831  0.2301
##  3 - 15    -0.5437 0.138 Inf -3.937  0.0072
##  4 - 5     -0.1957 0.134 Inf -1.456  0.9820
##  4 - 6     -0.2171 0.134 Inf -1.615  0.9557
##  4 - 7     -0.0602 0.134 Inf -0.448  1.0000
##  4 - 8     -0.2315 0.135 Inf -1.710  0.9305
##  4 - 9     -0.2740 0.137 Inf -2.003  0.7968
##  4 - 10    -0.2058 0.135 Inf -1.523  0.9732
##  4 - 11    -0.2755 0.137 Inf -2.010  0.7928
##  4 - 12    -0.3462 0.136 Inf -2.545  0.4076
##  4 - 13    -0.4184 0.137 Inf -3.046  0.1359
##  4 - 14    -0.1490 0.137 Inf -1.089  0.9991
##  4 - 15    -0.3000 0.137 Inf -2.186  0.6751
##  5 - 6     -0.0214 0.136 Inf -0.158  1.0000
##  5 - 7      0.1355 0.134 Inf  1.007  0.9996
##  5 - 8     -0.0359 0.135 Inf -0.266  1.0000
##  5 - 9     -0.0783 0.136 Inf -0.574  1.0000
##  5 - 10    -0.0101 0.136 Inf -0.074  1.0000
##  5 - 11    -0.0798 0.137 Inf -0.584  1.0000
##  5 - 12    -0.1506 0.137 Inf -1.103  0.9989
##  5 - 13    -0.2227 0.138 Inf -1.611  0.9568
##  5 - 14     0.0466 0.137 Inf  0.340  1.0000
##  5 - 15    -0.1044 0.137 Inf -0.760  1.0000
##  6 - 7      0.1569 0.135 Inf  1.160  0.9981
##  6 - 8     -0.0144 0.136 Inf -0.106  1.0000
##  6 - 9     -0.0569 0.138 Inf -0.413  1.0000
##  6 - 10     0.0113 0.136 Inf  0.083  1.0000
##  6 - 11    -0.0584 0.138 Inf -0.423  1.0000
##  6 - 12    -0.1291 0.137 Inf -0.941  0.9998
##  6 - 13    -0.2013 0.138 Inf -1.456  0.9820
##  6 - 14     0.0681 0.138 Inf  0.493  1.0000
##  6 - 15    -0.0829 0.139 Inf -0.599  1.0000
##  7 - 8     -0.1714 0.136 Inf -1.262  0.9955
##  7 - 9     -0.2138 0.136 Inf -1.568  0.9654
##  7 - 10    -0.1456 0.135 Inf -1.078  0.9992
##  7 - 11    -0.2153 0.136 Inf -1.579  0.9634
##  7 - 12    -0.2860 0.136 Inf -2.101  0.7347
##  7 - 13    -0.3582 0.137 Inf -2.608  0.3642
##  7 - 14    -0.0888 0.138 Inf -0.644  1.0000
##  7 - 15    -0.2399 0.138 Inf -1.743  0.9197
##  8 - 9     -0.0424 0.137 Inf -0.310  1.0000
##  8 - 10     0.0258 0.137 Inf  0.189  1.0000
##  8 - 11    -0.0439 0.138 Inf -0.319  1.0000
##  8 - 12    -0.1147 0.136 Inf -0.841  1.0000
##  8 - 13    -0.1868 0.138 Inf -1.352  0.9911
```

```
##  8 - 14     0.0825 0.138 Inf  0.597  1.0000
##  8 - 15    -0.0685 0.138 Inf -0.496  1.0000
##  9 - 10     0.0682 0.137 Inf  0.499  1.0000
##  9 - 11    -0.0015 0.138 Inf -0.011  1.0000
##  9 - 12    -0.0722 0.137 Inf -0.526  1.0000
##  9 - 13    -0.1444 0.139 Inf -1.039  0.9994
##  9 - 14     0.1250 0.139 Inf  0.900  0.9999
##  9 - 15    -0.0260 0.139 Inf -0.187  1.0000
##  10 - 11   -0.0697 0.137 Inf -0.508  1.0000
##  10 - 12   -0.1405 0.136 Inf -1.030  0.9995
##  10 - 13   -0.2126 0.138 Inf -1.543  0.9700
##  10 - 14    0.0567 0.138 Inf  0.411  1.0000
##  10 - 15   -0.0943 0.138 Inf -0.684  1.0000
##  11 - 12   -0.0708 0.138 Inf -0.514  1.0000
##  11 - 13   -0.1429 0.140 Inf -1.023  0.9995
##  11 - 14    0.1264 0.139 Inf  0.908  0.9999
##  11 - 15   -0.0246 0.139 Inf -0.177  1.0000
##  12 - 13   -0.0721 0.138 Inf -0.521  1.0000
##  12 - 14    0.1972 0.138 Inf  1.432  0.9846
##  12 - 15    0.0462 0.138 Inf  0.334  1.0000
##  13 - 14    0.2693 0.139 Inf  1.931  0.8378
##  13 - 15    0.1183 0.140 Inf  0.847  1.0000
##  14 - 15   -0.1510 0.139 Inf -1.084  0.9991
##
## Results are averaged over the levels of: BreedGroup, Play
## Results are given on the log (not the response) scale.
## P value adjustment: tukey method for comparing a family of 15 estimates
```

```
pairs(emmeans(EC_mod, ~BreedGroup))
```

```
##  contrast        estimate    SE  df z.ratio p.value
##  Coop - Mixed      0.0102 0.251 Inf 0.040   0.9991
##  Coop - Noncoop    1.0868 0.298 Inf 3.643   0.0008
##  Mixed - Noncoop   1.0766 0.273 Inf 3.949   0.0002
##
## Results are averaged over the levels of: Trial, Play
## Results are given on the log (not the response) scale.
## P value adjustment: tukey method for comparing a family of 3 estimates
```
